# Supplementary material for: What motivates people with type 2 diabetes to maintain lifestyle changes and what challenges do they experience? A qualitative evidence synthesis
Source: PLoS One. 2025 Sep 18;20(9):e0332276. doi: 10.1371/journal.pone.0332276 (PMC12445501; doi:10.1371/journal.pone.0332276)
Supplement: S1 Appendix — (DOCX) [file pone.0332276.s001.docx]

**S1 Appendix. The ENTREQ Checklist**

| **Nr** | **Item** | **Guide and description** | **Page** |
| --- | --- | --- | --- |
| 1 | Aim | To explore what motivates people with type 2 diabetes to maintain lifestyle changes over time and the challenges they experience. | Abstract,  p. 2 |
| 2 | Synthesis methodology | We used a thematic synthesis approach by Thomas & Harden. | p. 8 |
| 3 | Approach to searching | The search was comprehensive and pre-planned. | p. 6-7 |
| 4 | Inclusion criteria | We included studies of participants over 18 years of age with T2D who had managed to complete a lifestyle change for a minimum of 12 months. We included studies that used both qualitative methods for data collection and data analysis. | p. 7 |
| 5 | Data sources | We searched MEDLINE, CINAHL, PsycINFO and EMBASE from inception to 4 November 2024. We conducted reference checking and citation searches. | p. 6 |
| 6 | Electronic Search strategy | We developed a search strategy for MEDLINE, using subject headings and text words related to diabetes type 2 and lifestyle change, including concepts such as lifestyle, health behaviour, adherence to changes, weight reduction, diet, physical activity and smoking cessation. The search strategy was modified for the other databases. We used validated filters for identifying qualitative studies in each database | p. 6  S3 Appendix |
| 7 | Study screening methods | Two review authors (TB and NO, or CG and LVN) independently assessed the titles and abstracts to evaluate eligibility. We retrieved the full text of all the papers identified as potentially relevant by both review authors. Both review authors then assessed these papers independently. We resolved disagreements by discussion or, when required, by involving a third person (LVN or TB/NO) | p.7 |
| 8 | Study characteristics | The characteristics of the included studies are presented in Table 1. | p. 12-13  Table 1; and S6 Appendix |
| 9 | Study selection results | 4101 records screened.  128 reports assessed for eligibility in fulltext.  115 reports excluded.  13 reports of 12 studies included. | p. 11  Fig.1  S5 Appendix |
| 10 | Rationale for appraisal | We used a list of criteria that has been used in previous qualitative evidence syntheses. This list of criteria was originally based on the Critical Appraisal Skills Programme (CASP) tool but has since gone through several iterations. | p. 8  Table 2 (p. 14)  S6 Appendix |
| 11 | Appraisal items | The items were:   1. Setting and context is described sufficiently. 2. Selection strategy is described and appropriate. 3. Data collection strategy is described and justified. 4. Data analysis is described and appropriate. 5. The claims/findings are supported by sufficient evidence. 6. There is proof of reflexivity. 7. The study shows sensitivity to ethical concerns 8. There are other concerns. | Table 2 (p. 14) |
| 12 | Appraisal process | Two review authors (TB, NO) independently assessed methodological limitations for each study. We resolved disagreements by discussion or, when required, by involving a third person (LVN) | p. 8 |
| 13 | Appraisal result | We found poor reporting of researcher reflexivity across many of the studies. All studies gave some description, although sometimes very brief, of the context, participants, sampling, methods, and analysis. We assessed nine studies to have minor limitations, and three studies had minor to moderate limitations | s. 14  Table 2  S6 Appendix |
| 14 | Data extraction | Two review authors (TB, NO) extracted information about the included studies, including the objectives of the study, information about participants, setting, context, method, results, and author's conclusion. The study findings were extracted by TB and NO independently. Any discrepancies were discussed until we reached agreement. | p. 7-8 |
| 15 | Software | DistillerSR | p. 7 |
| 16 | Number of reviewers | Two reviewers were involved in coding and analysis. | p. 8-9 |
| 17 | Coding | In phase 1 we coded the result section of each article line-by-line. In phase 2, through consensus, we looked for similarities between the codes and structured them into descriptive themes according to their meaning and content. In phase 3, we organized the descriptive themes into analytical themes. | p. 8 |
| 18 | Study  Comparison | We divided the descriptive themes into two categories: 1. motivating factors and 2. challenges. Second, based on the locus of control theory we categorised the descriptive themes as factors that people appeared to perceive because of their own decisions or actions, or as beyond their control. | p. 8  Fig.2 |
| 19 | Derivation of  themes | The process of deriving the themes or constructs was inductive. | p. 8 |
| 20 | Quotations | Quotations from the primary studies are provided throughout the results. | s. 16 - 30 |
| 21 | Synthesis  output | We used our findings to develop a model using the locus of control theory and to develop implications for practice. | S. 30 - 31  Fig.2  Table 3 (p. 33-34) |
