# Supplementary material for: What motivates people with type 2 diabetes to maintain lifestyle changes and what challenges do they experience? A qualitative evidence synthesis
Source: PLoS One. 2025 Sep 18;20(9):e0332276. doi: 10.1371/journal.pone.0332276 (PMC12445501; doi:10.1371/journal.pone.0332276)
Supplement: S3 Appendix — (DOCX) [file pone.0332276.s003.docx]

**S3 Appendix. Search strategies.**

**Ovid MEDLINE**(R) ALL <1946 to November 04, 2024>

| 1 | Diabetes Mellitus, Type 2/ | 184666 |
| --- | --- | --- |
| 2 | (diabetes adj2 (type 2 or type ii)).tw. | 194621 |
| 3 | non insulin dependent.tw. | 11068 |
| 4 | (T2D or T2DM or DM2 or DM 2 or NIDDM).tw. | 67544 |
| 5 | ((adult onset or ketosis resistant) adj diabetes).tw. | 517 |
| 6 | or/1-5 | 259245 |
| 7 | Life Change/ or Healthy Lifestyle/ or Life Style Events/ | 8806 |
| 8 | Health Behavior/ or "Attitude to Health"/ or " Health Knowledge, Attitudes, Practice"/ | 255529 |
| 9 | "Treatment Adherence and Compliance"/ or "Patient Acceptance of Health Care"/ or Patient Compliance/ or Medication Adherence/ or Patient Participation/ | 171923 |
| 10 | (adherence or compliance).tw. | 311775 |
| 11 | Diet/ or Diet, Diabetic/ or Diet, Healthy/ | 206886 |
| 12 | ((health* or change*) adj4 (diet? or dietary or eat or eating or nutrition*)).tw. | 121981 |
| 13 | Weight Loss/ | 46328 |
| 14 | (weight? adj2 (loss or lose or losing or decreas* or reduc* or watch* or control*)).tw. | 174860 |
| 15 | exp Exercise/ or Physical Fitness/ | 282367 |
| 16 | (exercis* or physical activit*).tw. | 508012 |
| 17 | Smoking Cessation/ or Smoking Reduction/ | 33977 |
| 18 | (smoking adj2 (cessat* or quit* or stop* or g#ve up or giving up or abstin* or dehabituat* or reduc* or decreas*)).tw. | 46457 |
| 19 | or/7-18 | 1667331 |
| 20 | Qualitative Research/ | 94584 |
| 21 | Interviews as Topic/ | 70296 |
| 22 | Focus Groups/ | 39006 |
| 23 | Narration/ | 10878 |
| 24 | ((semi structured or semistructured or unstructured or informal or in depth or indepth or "face to face" or structured or guide) adj3 (interview* or discussion* or questionnaire*)).tw. | 202764 |
| 25 | (focus group* or qualitative or ethnograph* or fieldwork or field work or key informant).tw. | 411982 |
| 26 | or/20-25 | 580597 |
| 27 | 6 and 19 and 26 | 2490 |

**Ovid Embase** <1974 to 2024 November 04>

| 1 | Non insulin dependent diabetes mellitus/ | 369732 |
| --- | --- | --- |
| 2 | (diabetes adj2 (type 2 or type ii)).tw. | 301155 |
| 3 | non insulin dependent.tw. | 13041 |
| 4 | (T2D or T2DM or DM2 or DM 2 or NIDDM).tw. | 112080 |
| 5 | ((adult onset or ketosis resistant) adj diabetes).tw. | 699 |
| 6 | or/1-5 | 436299 |
| 7 | Life Style/ or Healthy Lifestyle/ or Life Event/ | 175970 |
| 8 | Health Behavior/ or "Attitude to Health"/ | 213660 |
| 9 | exp Patient Compliance/ or Patient Participation/ | 243205 |
| 10 | (adherence or compliance).tw. | 485040 |
| 11 | Diet/ or Healthy Diet/ or Diabetic Diet/ | 277916 |
| 12 | ((health* or change*) adj4 (diet? or dietary or eat or eating or nutrition*)).tw. | 155572 |
| 13 | exp Body Weight Loss/ | 109385 |
| 14 | (weight? adj2 (loss or lose or losing or decreas* or reduc* or watch* or control*)).tw. | 271193 |
| 15 | exp Exercise/ or Fitness/ | 496970 |
| 16 | (exercis* or physical activit*).tw. | 677694 |
| 17 | Smoking Cessation/ or Smoking Reduction/ | 73408 |
| 18 | (smoking adj2 (cessat* or quit* or stop* or g#ve up or giving up or abstin* or dehabituat* or reduc* or decreas*)).tw. | 61636 |
| 19 | or/7-18 | 2318984 |
| 20 | (("semi-structured" or semistructured or unstructured or informal or "in-depth" or indepth or "face-to-face" or structured or guide) adj3 (interview* or discussion* or questionnaire*)).ti,ab. or (focus group* or qualitative or ethnograph* or fieldwork or "field work" or "key informant").tw,kw. or Qualitative Research/ | 669061 |
| 21 | 6 and 19 and 20 | 3316 |
| 22 | limit 21 to embase | 1648 |

**CINAHL** <1981 - >

| S1 | (MH «Diabetes Mellitus, Type 2») | 72243 |
| --- | --- | --- |
| S2 | TI (diabetes N1 (type 2 or type ii) OR AB (diabetes N3 (type 2 or type ii) | 65799 |
| S3 | TI (non insulin dependent) OR AB (non insulin dependent) | 1435 |
| S4 | TI (T2D or T2DM or DM2 or DM 2 or NIDDM) OR AB (T2D or T2DM or DM2 or DM 2 or NIDDM) | 17850 |
| S5 | TI ((adult onset or ketosis resistant) N1 diabetes) OR AB ((adult onset or ketosis resistant) N1 diabetes) | 242 |
| S6 | S1 OR S2 OR S3 OR S4 OR S5 | 95527 |
| S7 | (MH “Life Style”) OR (“Life Change Event”) OR (MH “Life Style Changes”) | 50913 |
| S8 | TI ((health* or change*) N3 (behavio#r* or life style* or lifestyle*)) ) OR AB ( ((health* or change*) N# (behavio#r* or life style* or lifestyle*)) | 25143 |
| S9 | (MH "Attitude to Health") OR (MH "Health Beliefs") OR (MH "Attitude to Obesity") OR (MH "Patient Attitudes") OR (MH "Health Behavior") | 179187 |
| S10 | (MH “Patient Compliance+”) | 59772 |
| S11 | TI (adherence or compliance) OR AB (adherence or compliance) | 107330 |
| S12 | (MH “Diet”) OR (MH “Diabetic diet”) | 67091 |
| S13 | TI ((health* or change*) N3 (diet? or dietary or eat or eating or nutrition*)) OR AB ((health* or change*) N3 (diet? or dietary or eat or eating or nutrition*)) | 50825 |
| S14 | (MH "Weight Loss") OR (MH "Weight Control") | 33618 |
| S15 | TI (weight? N1 (loss or lose or losing or decreas* or reduc* or watch* or control*)) OR AB (weight? N1 (loss or lose or losing or decreas* or reduc* or watch* or control*)) | 43172 |
| S16 | MH (“Exercise+”) OR (MH “Physical fitness”) | 146642 |
| S17 | TI (exercis* or physical activit*) OR AB (exercis* or physical activit*) | 211369 |
| S18 | (MH «Smoking Cessation») | 23355 |
| S19 | TI (smoking N1 (cessat* or quit* or stop* or give up or giving up or abstin* or dehabituat* or reduc* or decreas*)) OR AB (smoking N1 (cessat* or quit* or stop* or g#ve up or giving up or abstin* or dehabituat* or reduc* or decreas*)) | 21294 |
| S20 | S7 OR S8 OR S9 OR S10 OR S11 OR S12 OR S13 OR S14 OR S15 OR S16 OR S17 OR S18 OR S19 | 733514 |
| S21 | TI (interview) OR AB (interview) | 231176 |
| S22 | (MH “Audiorecording”) | 48768 |
| S23 | (MH “Qualitative Studies”) | 157589 |
| S24 | TI qualitative stud* OR AB qualitative stud* | 91660 |
| S25 | S21 OR S22 OR S23 OR S24 | 345734 |
| S26 | S6 AND S20 AND S25 | 1727 |
|  | S6 AND S20 AND S25 -Exclude MEDLINE records | 1012 |

**APA PsycInfo** <1806 to October 2024 Week 4>

| 1 | Type 2 Diabetes/ | 5078 |
| --- | --- | --- |
| 2 | (diabetes adj2 (type 2 or type ii)).tw. | 10412 |
| 3 | non insulin dependent.tw. | 227 |
| 4 | (T2D or T2DM or DM2 or DM 2 or NIDDM).tw. | 2675 |
| 5 | ((adult onset or ketosis resistant) adj diabetes).tw. | 38 |
| 6 | or/1-5 | 10844 |
| 7 | exp Lifestyle/ or Life Changes/ | 20758 |
| 8 | Health Behavior/ or Preventive Health Behavior/ or Self-Care/ or Health Attitude/ or Health Knowlegde/ | 37578 |
| 9 | Treatment Compliance/ or Client Attitudes/ or Client participation/ | 41583 |
| 10 | (adherence or compliance).tw. | 67371 |
| 11 | exp Diets/ | 21597 |
| 12 | ((health* or change*) adj4 (diet? or dietary or eat or eating or nutrition*)).tw. | 21389 |
| 13 | Weight Loss/ | 4931 |
| 14 | (weight? adj2 (loss or lose or losing or decreas* or reduc* or watch* or control*)).tw. | 21894 |
| 15 | exp Exercise/ or Physical Fitness/ | 37975 |
| 16 | (exercis* or physical activit*).tw. | 122666 |
| 17 | exp Smoking Cessation/ | 15584 |
| 18 | (smoking adj2 (cessat* or quit* or stop* or g#ve up or giving up or abstin* or dehabituat* or reduc* or decreas*)).tw. | 19828 |
| 19 | or/7-18 | 312850 |
| 20 | (experiences or interview* or qualitative).tw. | 770213 |
| 21 | 6 and 19 and 20 | 830 |
| 22 | limit 21 to "remove medline records" | 505 |
