# Supplementary material for: What motivates people with type 2 diabetes to maintain lifestyle changes and what challenges do they experience? A qualitative evidence synthesis
Source: PLoS One. 2025 Sep 18;20(9):e0332276. doi: 10.1371/journal.pone.0332276 (PMC12445501; doi:10.1371/journal.pone.0332276)
Supplement: S5 Appendix — (DOCX) [file pone.0332276.s005.docx]

**S5 Appendix. Table of excluded studies**

| Study | **Reason for exclusion** |
| --- | --- |
| Abbott S, Gunnell C. Are older people with diabetes compliant or empowered? Pract Nurs. 2005; 16. doi: 10.12968/pnur.2005.16.11.19975. | Excluded because of topic of interest. The main topic of interest was the experiences of receiving diabetes services in older adults with T2D, their understanding of self-management, and their values and preferences, rather than maintaining lifestyle changes over time. |
| Advika, TS, Idiculla, J, Kumari, SJ. Exercise in patients with type 2 diabetes: facilitators and barriers - a qualitative study. J Family Med Prim Care, 2017; 6: 288-292. doi: 10.4103/2249-4863.219998 | Excluded because of topic of interest. Although the study explored motivations and barriers to exercise in adults with T2D, the focus was not on maintenance of exercise over time. |
| Aghamohammadi-Kalkhoran M, Valizadeh S, Mohammadi E, Ebrahimi H, Karimollahi M. Health according to the experiences of Iranian women with diabetes: a phenomenological study. Nurs Health Sci. 2012; 14: 285-291. doi: 10.1111/j.1442-2018.2011.00672.x. | Excluded because of topic of interest. The main topic of interest was T2B patients’ perceptions on the meaning of health and not maintenance of lifestyle changes over time. |
| Ahlin K, Billhult A. Lifestyle changes – a continuous, inner struggle for women with type 2 diabetes: A qualitative study. Scand J Prim Health Care, 2012; 30: 41–47. doi: 10.3109/02813432.2011.654193 | Excluded because of topic of interest. Although the study explored how adult women with T2D handle necessary lifestyle changes it does not focus on maintenance of changes over time. |
| Akhter K, Bunn C, Graffy J, Donald S, Ward C, Simmons D. Empowerment-based education for established type 2 diabetes in rural England. Pract Diab, 2017; 34: 83-88. doi: 10.1002/pdi.2088 | Excluded because of topic of interest. The main topic of interest in the qualitative arm of the study was the acceptability and usefulness of an 3.5-hour workshop about self-care in adults with T2D and not maintenance of lifestyle changes over time. |
| Anitha Rani M, Shriraam V. Are patients with type 2 diabetes not aware or are they unable to practice self-care? A qualitative study in rural South India. J Prim Care Community Health, 2019; 10: 2150132719865820. doi: 10.1177/2150132719865820 | Excluded because of topic of interest. The main focus was on perceived barriers to self-care among adults with T2B, rather than on barriers encountered when maintaining lifestyle changes over time. |
| Ansari RM, Harris MF, Hosseinzadeh H, Zwar N. Applications of a chronic care model for self-management of type 2 diabetes: a qualitative analysis. Int J Environ Res Public Health, 2021; 18: 10840. doi: 10.3390/ijerph182010840. | Excluded because of topic of interest. The main topic of interest was self-management of T2D but not maintaining lifestyle changes over time. |
| Arana, MA, Valderas JM, Solomon J. Being tested but not educated - a qualitative focus group study exploring patients' perceptions of diabetic dietary advice. BMC Fam Pract, 2019; 20. doi: 10.1186/s12875-018-0892-5. | Excluded because of topic of interest. Although the main topic was experience with receiving dietary advice aomong adults with T2D and barriers faced in diet management, the focus was not on maintenance of dietary changes over time. |
| Areshtanab HN, Moonaghi HK, Jouybari L, Zamanzadeh V, Ebrahimi H. Adapting life to the reality of diabetes. East Mediterr Health J, 2018; 24: 729-735. doi: 10.26719/2018.24.8.729. | Excluded because of topic of interest. The main focus was experiences in adaptation to T2D in adult patients and not maintenance of lifestyle changes over time. |
| Baptista S, Wadley G, Bird D, Oldenburg B, Speight J, The My Diabetes Coach Research Group. Acceptability of an embodied conversational agent for type 2 diabetes self-management education and support via a smartphone app: mixed methods study. JMIR Mhealth Uhealth, 2020; 8 :e17038. doi: 10.2196/17038. | Excluded because of topic of interest. The main focus was on the acceptability and perceived support of an embodied conversational agent in a diabetes self-management education app. It did not focus on maintaining lifestyle changes over time. |
| Blicher-Hansen J, Chilcot J, Gardner B. Experiences of successful physical activity maintenance among adults with type 2 diabetes: a theory-based qualitative study. Psychol Health, 2024; 39(3): 399-416. doi: 10.1080/08870446.2022.2063863. | Excluded because of topic of interest. Long-term maintenance was defined as sustaining physical activity for at least six months rather than 12 months or more. |
| Boocock RC, Haste A, Moore HJ, Lake AA. Barriers and enablers to engagement with a type 2 diabetes remission project in the North East of England: Qualitative perspectives of patients. J Nutr Sci, 2024, 13: e28. doi: 10.1017/jns.2024.30 | Excluded because of topic of interest. The study explored and external barriers and enablers to adherence to a 12-week dietary and physical activity intervention. There was no indication that participants had maintained life style changes for a longer period of time. |
| Burns D, Skelly AH. African American women with type 2 diabetes: meeting the daily challenges of self care. J Multicult Nurs Health, 2005; 11: 6-10. | Excluded because of topic of interest. The main topic of interest is the daily challenges and experiences of self-management in adult women with TB2 and not maintenance of lifestyle changes over time. |
| Carolan M, Holman J, Ferrari M. Experiences of diabetes self-management: a focus group study among Australians with type 2 diabetes. J Clin Nurs, 2014; 24:1011-23. doi: 10.1111/jocn.12724. | Excluded because of topic of interest.  Although the study explored adult T2D patients’ experiences with self-management the focus was not on maintenance of lifestyle changes over time. |
| Carolan-Olah M, Cassa, A. The experiences of older Italian migrants with type 2 diabetes: a qualitative study. J Transcult Nurs, 2018; 29: 172-179. doi: 10.1177/1043659617696974. | Excluded because of topic of interest. The main topic of interest was T2D patients’ experience of living with diabetes and accessing care and not maintenance of lifestyle changes over time. |
| Carpenter RD. Challenges and movement to resolution for persons living with diabetes. Arch Psychiatr Nurs, 2014; 28: 352-3. doi: 10.1016/j.apnu.2014.06.002. | Excluded because of topic of interest. The main topic of interest was T2D patients’ experience of the dimensions of diabetes in general and approaches to resolve it and not maintenance of lifestyle changes over time. |
| da Silva Rocha JM, Moreira TM, Rodrigues DP. Adaptation of the patient suffering from diabetes mellitus type 2 to the disease and to the treatment. Rev Rene, 2005; 6. | Excluded because of topic of interest. Although the study explored adult T2D patients’ adaptation to treatment (e.g. exercise and diet) the focus was not on maintenance of lifestyle changes over time. |
| Desse TA, Namara KM, Manias E. Patient-perceived challenges to type 2 diabetes self-management in Sub-Saharan Africa: a qualitative exploratory study. Sci Diabetes Self Manag Care, 2024; 1-13. doi: 10.1177/26350106241279809 | Excluded because of topic of interest. The main topic of interest were challenges influencing self-management in T2D patients. The participants had been treated for their diabetes for at least one year but it is unclear whether the challenges described were related to maintaining lifestyle changes. The findings indicate the opposite. |
| Díaz de León-Castañeda C. Experiencias sobre el desarrollo del autocuidado en pacientes con diabetes tipo 2 integrantes de un "Grupo de Ayuda Mutua" y atendidos por un equipo interdisciplinario. Revista de la ALAD. 2023; 13(2): 50-61. | Excluded because of topic of interest. The main topic of interest was T2D patients experiences regarding the development of knowledge, skills and self-care behaviors. Although facilitators and barriers to maintaining self-care is explored the focus is not on maintenance over time. |
| Dietrich UC. Factors influencing the attitudes held by women with type II diabetes: a qualitative study. Patient Educ Couns, 1996; 29: 13-23. doi: 10.1016/0738-3991(96)00930-5. | Excluded because of topic of interest. The main focus was adult T2D patients’ attitudes towards diabetes and its treatment in general and not maintenance of lifestyle changes over time. |
| Dimova ED, Swanson V, Evans JM. Gender and diet management in type 2 diabetes. Chronic Illn, 2021; 17: 362-376. doi: 10.1177/1742395319873375. | Excluded because of topic of interest. The main topic of interest was the experiences of diet management among adults with a recent T2D and not maintenance of lifestyle choices over time. |
| Du Q-H, Zhang Z-C, Yang Y, Luo X-X, Liu L, Jia H-H. How health seeking behavior develops in patients with type 2 diabetes: a qualitative study based on health belief model in China. Front Public Health, 2024; 12: 1414903. doi: 10.3389/fpubh.2024.1414903 | Excluded because of topic of interest. The study explores health seeking behaviour in patients with T2D. Although participants had been diagnosed with T2D for more than two years it is unclear whether they have maintained "health seeking behaviours" for at least one year |
| Dye CJ, Haley-Zitlin V, Willoughby D. Insights from older adults with type 2 diabetes: making dietary and exercise changes. Diabetes Educ, 2003; 29: 116-27. doi: 10.1177/014572170302900116. | Excluded because of topic of interest. The main topic of interest was the experiences of making changes in diet and exercise among older adults with T2D but not maintenance of these changes over time. |
| Early KB, Shultz JA, Corbett C. Assessing diabetes dietary goals and self-management based on in-depth interviews with Latino and Caucasian clients with type 2 diabetes. J Transcult Nurs, 2009; 20: 371-81. doi: 10.1177/1043659609334928 | Excluded because of topic of interest. This main focus was the self-management experiences of adults with T2D and not maintenance of lifestyle changes over time. |
| Eborall HC, Virdee SK, Patel N, Redwood S, Greenfield SM, Stone MA. "And now for the good news..." the impact of negative and positive messages in self-management education for people with Type 2 diabetes: A qualitative study in an ethnically diverse population. Chronic Illn, 2016; 12:3-17. doi: 10.1177/1742395315577965. | Excluded because of topic of interest. The main topic of interest was the impact of a specific education programme on adults with T2D and not maintenance of lifestyle changes over time. |
| Ellison GC, Rayman KM. Exemplars' experience of self-managing type 2 diabetes. Diabetes Educ, 1998; 24: 325-30. doi: 10.1177/014572179802400307. | Excluded because of topic of interest. The main topic of interest was the experiences of self-management among adults with T2D and not maintenance of lifestyle changes over time. |
| Eustace A. Perspectives on adherence: Tobagonian women with T perspectives on adherence: Tobagonian women with type 2 diabetes. PhD Thesis. | Excluded because of topic of interest. Although findings include both enablers and barriers to adherence to treatment, including life style changes, among Tobagonian women diagnosed with T2D for at least one year. However, we do not know if patients had maintained changes for at least one year. |
| Fitzpatrick SL, Hill-Briggs F. Strategies for sustained weight management: perspectives from African American patients with type 2 diabetes. Diabetes Educ, 2017; 43: 304-310. doi: 10.1177/0145721717699071 | Excluded because of topic of interest. The main topic of interest was opinions about strategies for sustained weight management among adults with T2D who attended group interviews three months after a 18-20 week trial intervention. It does not focus on maintenance of lifestyle changes over time. |
| Frandsen KB, Kristensen JS. Diet and lifestyle in type 2 diabetes: the patient's perspective. Prac Diabetes Int, 2002; 19: 77-80. doi: 10.1002/pdi.327 | Excluded because of topic of interest. The main topic of interest were motivators and barriers relating to diet and lifestyle changes in adults with T2D but not on maintenance of these changes over time. |
| Fritschi C, Martyn-Nemeth P, Zhu B, Jung Kim M. Active learning: lessons from women with type 2 diabetes in a walking program. Diabetes Educ, 2019; 45: 370-379. doi: 10.1177/0145721719848437. | Excluded because of topic of interest. The main topic of interest was the experiences of adult T2D patients who had recently completed a 12 week exercise programme and not maintenance of lifestyle changes over time. |
| Gardsten C, Blomqvist K, Rask M, Larsson A, Lindberg A, Olsson G. Challenges in everyday life among recently diagnosed and more experienced adults with type 2 diabetes: a multistage focus group study. J Clin Nurs, 2018; 27: 3666-3678. doi: 10.1111/jocn.14330. | Excluded because of topic of interest. The main focus of the study was the everyday life challenges faced by adults with T2D and not maintenance of lifestyle changes over time. |
| Gentsch AT, Reed MK, Cunningham A, Chang AM, Kahn S, Kovalsky D, et al. "Once I take that one bite": the consideration of harm reduction as a strategy to support dietary change for patients with diabetes. BMC Endocr Disord, 2024; 24(1): 1-9. doi: 10.1186/s12902-023-01529-6 | Excluded because topic of interest and participants. The study explored barriers to maintaining a healthy diet in patients with T1D and T2D but did not consider long-term maintenance. Additionally, it is not possible to separate the findings between T2D and T1D patients. |
| Gibson R, D'Annibale M, Oliver N, McGowan B, Forbes G, Crayton E, et al. Exploration of the individual, social and environmental factors influencing dietary behaviour in shift workers with type 2 diabetes working in UK healthcare-The Shift-Diabetes Study: A qualitative study using the theoretical domains framework. Diabet Med, 2024; 41(2): e15179. doi: 10.1111/dme.15179 | Excluded because of topic of interest. The study examined factors affecting dietary behavior in shift workers with T2D for an average of seven years. It identified both facilitators and barriers to healthy eating, but it's unclear if participants maintained these behaviors for over a year. |
| Girling LM. The subjective construction of disease control among older adults with type II diabetes. PhD Thesis. | Excluded because of topic of interest. The main focus was on diabetic adults’ understanding of disease control and not on maintaining lifestyle changes over time. |
| Girling LM, Chard SE, Eckert JK. Ascribed meaning of disease control: perspectives of patients with type 2 diabetes. J Patient Exp, 2018; 5: 160–166. doi: 10.1177/2374373517745915. | Excluded because of topic of interest. This study explored how older adults with T2D conceptua-lise diabetes control. The focus of the study was not on maintenance of lifestyle changes over time. |
| Graue M, Igland J, Haugstvedt A, Hernar I, Birkeland KI, Zoffmann V, et al. Evaluation of an interprofessional follow-up intervention among people with type 2 diabetes in primary care. A randomized controlled trial with embedded qualitative interviews. PLOS ONE, 2023;18: e0291255. doi: 10.1371/journal.pone.0291255 | Excluded because of topic of interest. This qualitative study, part of an RCT, involved people with T2D attending a 12-month behavioral, empowerment-based intervention. It is unclear if they sustained lifestyle changes during this period, as interviews focused on their experience with the intervention rather than on maintaining lifestyle changes. |
| Grech J, Norman IJ, Sammut R. Exploring the smoking cessation needs of individuals with diabetes using the Information-Motivation-Behavior Skills model. Tob Prev Cessation, 2024;10. doi: 10.18332/tpc/181366 | Excluded because of participants. The study aimed to identify the needs of individuals with type 1 and type 2 diabetes to quit smoking. Findings can be separated between current and former smokers (mean 2 years since cessation), but not between T2D and T1D. |
| Gumber, L. Knowledge gaps and other barriers in type 2 diabetes management: Findings from interviews with South Asian women. Diabetes Prim Care, 2014; 16: 86-91. | Excluded because of topic of interest. This study explored whether there is a knowledge gap among adults with T2D. The focus of the study was not maintenance of lifestyle changes over time. |
| Gutschall M, Onega LL, Wright WK. Patients' perspectives about dietary maintenance in type 2 diabetes. Top Clin Nutr, 2011; 26: 180-189. doi: 10.1097/TIN.0b013e3182260d69 | Excluded because of topic of interest. Although this study explored adult T2D patients’ perspectives about dietary maintenance the duration of their maintenance is unclear. |
| Habte, BM, Kebede T, Fenta TG, Boon H. Barriers and facilitators to adherence to anti-diabetic medications: Ethiopian patients' perspectives. Afr J Prim Health Care Fam Med, 2017; 9: 1411. doi: 10.4102/phcfm.v9i1.1411 | Excluded because of topic of interest. The focus of this topic was on barriers and facilitators to anti-diabetic medications, and not on the maintenance of lifestyle changes. |
| He W, Zhang Y, Zhao F. Factors influencing exercises in Chinese people with type 2 diabetes. Int Nurs Rev, 2013; 60:494-500. doi: 10.1111/inr.12046. | Excluded because of topic of interest. Although the study explored perspectives about adherence to exercise among adults with T2D, the focus was not on maintenance of lifestyle changes over time. |
| Iqbal S, Iqbal H, Kagan C. Intergenerational differences in healthy eating beliefs among British Pakistanis with type 2 diabetes. Diabet Med, 2024; 41(4): e15222. doi: 10.1111/dme.15222 | Excluded because of topic of interest. The study explores beliefs about healthy eating and food practices in British Pakistanis with T2D, but it lacks information on how long participants had T2D or if they maintained healthy eating habits for over a year. |
| Jeanfreau, SG. A grounded theory study of transitions toward self-management of type 2 diabetes as experienced by adults. PhD Thesis. Louisiana State University | Excluded because of topic of interest. The study focused on transitions toward self-management in adults with T2D and not maintenance of lifestyle changes over time. |
| Jordan OJ, Benitez A, Burnet DL, Quinn MT, Baig AA. The role of family in diabetes management for Mexican American Adults. Hisp Health Care Int, 2024; 22(2): 109-18. doi: 10.1177/15404153231206086 | Excluded because of topic of interest. The study explores how family influences self-management in Mexican Americans with T2D, high-lighting both challenges and facilitators. However, it is unclear if participants maintained lifestyle changes for over a year. |
| Jones L, Crabb S, Turnbull D, Oxlad M. Barriers and facilitators to effective type 2 diabetes management in a rural context: a qualitative study with diabetic patients and health professionals. J Health Psychol, 2014; 19: 441-53. doi: 10.1177/1359105312473786. | Excluded because of topic of interest. The study explored the views and experiences of adults with T2D about diabetes management in general and not maintenance of lifestyle changes over time. |
| Kang HJ, Wang JCK, Burns SF, Leow MK. Is self-determined motivation a useful agent to overcome perceived exercise barriers in patients with type 2 diabetes mellitus? Front Psychol, 2021; 12: 627815. doi: 10.3389/fpsyg.2021.627815. | Excluded because of topic of interest.  Although the study explored motivations and barriers to physical activity (PA) in adults with T2D, the focus was not on maintenance of PA over time. |
| Kapur K, Kapur A, Ramachandran S, Mohan V, Aravind SR, Badgandi M. et al. Barriers to changing dietary behavior. J Assoc Physicians India, 2008; 56: 27-32. | Excluded because of topic of interest. While the focus was on barriers to dietary changes among adults with T2D, it was not clear if those who managed to change their diet had maintained these changes for 12 months or longer. |
| Kheir N, Greer W, Yousif A, Al Geed H, Al Okkah R. Knowledge, attitude and practices of Qatari patients with type 2 diabetes mellitus. Int J Pharm Pract, 2011; 19: 185-191. doi: 10.1111/j.2042-7174.2011.00118.x. | Excluded because of study design. The study was a quantitative questionnaire study and not a qualitative study. |
| Knutsen IR, Foss C. Når de sier: Det kan ikke du spise, blir jeg arg! -- Å leve på den «rette» måten med diabetes type 2. [I get angry when somebody says that you can’t eat this: living the “right” way with type-2 diabetes]. Nordic Nurs Res, 2017; 7: 280–293. doi: 10.18261/issn.1892-2686-2017-04-0 | Excluded because of topic of interest. The study focused on adults with T2D who struggled with self-management and not maintenance of lifestyle changes over time. |
| Koch T, Kralik D, Taylor J. Men living with diabetes: minimizing the intrusiveness of the disease. J Clin Nurs, 2000; 9:247-254. doi: 10.1046/j.1365-2702.2000.00341.x. | Excluded because of topic of interest. The main focus was on living with T2D in general and not maintenance of lifestyle changes over time. |
| Kumar R, Mohammadnezhad M, Khan S. Perception of Type 2 Diabetes Mellitus (T2DM) patients on diabetes self-care management in Fiji. PLOS ONE, 2024;19(5): e0304708. | Excluded because of topic of interest. The study explored perceptions of T2D patients' self-care management, including those who attended clinic for at least 6 months. It is unclear if these patients maintained lifestyle changes for at least one year. |
| Lakshmi N, Anjana RM, Rhodes EC, Nora V, Rakesh H, Palmer CNA, et al. A qualitative study on perceptions and practices of diabetes prevention and management in rural South India. J Diabetol, 2023; 14(4): 239-47. doi: 10.4103/jod.jod_77_23. | Excluded because of topic of interest. The study explores management but not maintenance of lifestyle interventions in adults with T2D. Although participants had T2D for at least a year, it is unclear if they maintained lifestyle changes during this time, with findings suggesting otherwise. |
| Lan Z, Zhigang P, Fulai S. Self-management and family support among elderly patients with type 2 diabetes in community: based on semi-structured interviews. Chin J Gen Pract, 2024; 23(2): 126-31. | Excluded because of topic of interest. The main focus was the status of self-management and family support among elderly diabetic patients in community and not maintenance of lifestyle changes over time. |
| Laranjo L, Neves AL, Costa A, Ribeiro RT, Couto L, Sa AB. Facilitators, barriers and expectations in the self-management of type 2 diabetes: a qualitative study from Portugal. Eur J Gen Pract, 2015; 21:103-110. doi: 10.3109/13814788.2014.1000855. | Excluded because of topic of interest. Although the topic of interest is facilitators and barriers to self-management, the focus is not on maintenance of lifestyle changes over time. |
| Lawton J, Parry O, Peel E, Douglas M. Diabetes service provision: a qualitative study of newly diagnosed Type 2 diabetes patients' experiences and views. Diabet Med 2005; 22: 1246-1251. doi: 10.1111/j.1464-5491.2005.01619.x. | Excluded because of topic of interest. The main topic of interest was the views and experiences of diabetes services in adults recently diagnosed with T2D and not on maintenance of lifestyle changes over time. |
| Ledford CJW, Fulleborn ST, Jackson JT, Rogers T, Samar H. Dissonance in the discourse of the duration of diabetes: A mixed methods study of patient perceptions and clinical practice. Health Expect, 2021; 24: 1187–1196. doi: 10.1111/hex.13245 | Excluded because of topic of interest. This study explored how patients perceive the timeline of diabetes along the continuum of glycaemic control and their goals of care and not maintenance of lifestyle changes over time. |
| Lee M, Khoo HS, Krishnasamy C, Loo ME, Wong SKW, Cheng SC, et al. Experiences of living with overweight/ obesity and early type 2 diabetes in Singapore-a qualitative interview study. BMJ Open, 2024; 14(5): e079082. doi: 10.1136/bmjopen-2023-079082 | Excluded because of topic of interest. One aim of the study was to explore weight management in overweight/obese people with T2D. Although some successfully lost weight, it is unclear if they maintained a healthy lifestyle for over a year. |
| Lidegaard LP, Schwennesen N, Willaing I, Faerch K. Barriers to and motivators for physical activity among people with Type 2 diabetes: patients' perspectives. Diabet Med, 2016; 33:1677-1685. doi: 10.1111/dme.13167. | Excluded because topic of interest. Although the study explores motivators and barriers to physical activity in adults with T2D and the findings indicate that some patients exercise regularly, there is no information on whether they have maintained this exercise routine for 12 months or longer. |
| Liska J, Mical M, Maillard C, Dessapt C, Bendig E, Mai D, et al. Mapping the cardiometabolic patient experience and self-care behaviors to inform design, implementation, and persistent use of digital health care solutions: mixed methods study. JMIR Form Res, 2024; 8: e43683. doi: 10.2196/43683 | Excluded because topic of interest and. The study aimed to gain insights into self-care behaviors of individuals with cardiometabolic conditions, including T2D. However, it did not explore maintenance of self-care and lifestyle changes over time (> 1 year), and findings cannot be separated between T2D patients and those with acute coronary syndrome. |
| Lubaki J-PF, Francis JM, Omole OB. Perspectives for glycaemic control in type 2 diabetes in Kinshasa, Democratic Republic of the Congo. Health Promot Int, 2023; 38(5): 1-12. doi: 10.1093/heapro/daad128 | Excluded because of topic of interest. The study describes perspectives on glycemic control among people with T2D, with a mean diabetes duration of 5 years. About half had good glycemic control, but it is unclear if this resulted from maintaining lifestyle changes over time (> 1 year). |
| Lundberg PC, Thrakul S. Diabetes type 2 self-management among Thai Muslim women. J Nurs Healthcare Chron Illn, 2011; 3: 52-60. doi: 10.1111/j.1752-9824.2011.01079.x | Excluded because of topic of interest.  Although the study explores self-management in adults with T2D and the findings indicate that several patients engage in lifestyle changes such as dietary management and exercise, there is no information on whether these changes were maintained for 12 months or more. |
| Manser ST, Sekar P, Bonilla Z, Ford B, Shippee N, Busch AM, et al. Homelessness and type 2 diabetes: a qualitative study of facilitators and barriers to self-management and medication adherence. J Gen Intern Med, 2024. doi: 10.1007/s11606-024-09030-z | Excluded because of topic of interest. The study explored barriers and facilitators to medication adherence and self-management for people with T2D who experienced homelessness. While participants reported homelessness for at least one night in the past 12 months, it is unclear if they maintained lifestyle changes during this period. |
| Matpady P, Maiya AG, Saraswat PP, Rao CR, Pai MS, Anupama SD, et al. Barriers and enablers for physical activity engagement among individuals from India with type 2 diabetes mellitus: a mixed-method study. J Phys Act Health, 2024; 21(5): 519-27. doi: 10.1123/jpah.2023-0574 | Excluded because of topic of interest. The study addresses barriers and facilitatiors to physical activity in adults with TD2. However, there is no information about whether participants had maintained PA over time (> 1 yr). |
| Matpady P, Maiya AG, Saraswat PP, Mayya, SS, Pai, MS, Anupama DS et al. Dietary self-management practices among persons with T2DM: an exploratory qualitative study from western-coast of India. Diabetes Metab Syndr, 2020;14: 2161-2167. doi: 10.1016/j.dsx.2020.10.033. | Excluded because of topic of interest. Although the study explores dietary self-management practices, barriers, and enablers among adults with T2D, the maintenance of dietary changes over time is not explored. |
| McCarthy J, Psaros C, Wexler DJ, Delahanty LM. Medical nutrition therapy, in-person, or telephone group lifestyle intervention for type 2 diabetes? A qualitative study of patient perceptions and treatment references. Sci Diabetes Self Manag Care, 2024; 50(2): 130-40. doi: 10.1177/26350106241232635 | Excluded because of topic of interest. The study addresses preferences to different types of delivery prior to the launch of a two-year lifestyle intervention among people with T2D and not maintenance of lifestyle changes over time. |
| McCord EC, Brandenburg C. Beliefs and attitudes of persons with diabetes. Fam Med, 1995; 27: 267-71. | Excluded because of topic of interest. The study explores self-management of diabetes in 16 adults with T2D for at least 3 years. Nine are described as 'compliant' with lifestyle changes by their GPs, but the duration of compliance is unclear, and findings cannot be separated between compliant and non-compliant individuals. |
| McGloin H, Timmins F, Coates V, Boore J. A case study approach to the examination of a telephone-based health coaching intervention in facilitating behaviour change for adults with Type 2 diabetes. J Clin Nurs, 2015; 24: 1246-1257. doi: 10.1111/jocn.12692. | Excluded because of topic of interest. The qualitative arm of the study included people with T2D who had received telephone-based coaching to facilitate lifestyle changes. The study offers insights into lifestyle changes over 12 months but does not confirm if all participants maintained these changes throughout. |
| Mihalko SL, Cox P, Danhauer SC, Kirk JK, Black HL, Shumaker SA. Living with type 2 diabetes: A social cognitive perspective on adherence. Patient Educ Couns, 2024; 124: 108275. doi: 10.1016/j.pec.2024.108275 | Excluded because of topic of interest. This mixed-methods study explores the personal values motivating self-care behaviors in adults with T2D. While adherence to self-care behaviors is addressed, there is no information on the duration of adherence. |
| Mihwan K, Haejung L, Gaeun P, Ah Reum K. Participation experience in self-care program for type 2 diabetes: A mixed-methods study. J Korean Gerontol Nurs, 2024; 26(1): 31-42. doi: 10.17079/jkgn.2023.00220 | Excluded because of topic of interest. The study explored participation experiences of adult T2D patients attending a self-care programme. Long-term maintenance of lifestyle changes was defined as 6 months or more. |
| Mikkelsen TJ, Agerskov H, Jensen DM, Stenager E, Rothmann MJ. Living with schizophrenia and type 2 diabetes and the implication for diabetes self-care: a qualitative study. J Clin Nurs, 2024; 33(5): 1862-74. doi: 10.1111/jocn.17001 | Excluded because of topic of interest. The study aims to explore the challenges of self-management in adults with both schizophrenia and T2D, but it does not focus on the maintenance of lifestyle changes over time. |
| Mogre V, Johnson NA, Tzelepis F, Paul C. Barriers to diabetic self-care: A qualitative study of patients' and healthcare providers' perspectives. J Clin Nurs, 2019; 28: 2296-2308. doi: 10.1111/jocn.14835. | Excluded because of topic of interest. The main focus was perceived barriers to self-care in adults with T2D and not maintenance of lifestyle changes over time. |
| Mokoena RSN, Makhavhu EM, Tshivhase L. Understanding the struggle: Unique challenges of adherence in male diabetic patients in Tshwane. S Afr Fam Pract, 2024; 66(1): e1-e8. doi: 10.4102/safp.v66i1.5998 | Excluded because of topic of interest. The study explored treatment adherence in adults diagnosed with T2D for at least one year, focusing only on medication and not lifestyle changes. |
| Montilva-Monsalve J, Dimantas B, Perski O, Gutman LM. Barriers and enablers to the adoption of a healthier diet using an app: qualitative interview study with patients with type 2 diabetes mellitus. JMIR Diabet, 2023; 8: e49097. doi: 10.2196/49097 | Excluded because of topic of interest. The study explored enablers and barriers to adopting a healthier diet using a behavior change app in patients with recent (<12 mo) and long-standing (>18 mo) T2D diagnoses. Findings are separated between the two groups, but it is unclear if long-standing patients maintained healthy eating behaviors over time (> 1 yr). |
| Ntanda GM, Sia D, Tchouaket EN, Philibert L. Social determinants influencing the non-adoption of norms favorable to the prevention and control of type 2 diabetes: qualitative research. Inquiry, 2024; 61. doi: 10.1177/00469580241282051 | Excluded because of topic of interest. The study explores social determinants influencing the rejection or adoption of lifestyle changes (diet and physical activity) in adult migrants from sub-Saharan Africa at risk of or with T2D. Findings cannot be separated between those with T2D and those at risk, and it is unclear if those with T2D maintained lifestyle changes for over a year. |
| Odgers-Jewell K, Isenring EA, Thomas R, Reidlinger DP. Group participants' experiences of a patient-directed group-based education program for the management of type 2 diabetes mellitus. PLOS ONE, 2017; 12: e0177688. doi: 10.1371/journal.pone.0177688. | Excluded because of topic of interest. The main focus was the perceived impact of group interaction on motivation for self-management and not maintenance of lifestyle changes over time. |
| Oftedal B, Karlsen B, Bru E. Perceived support from healthcare practitioners among adults with type 2 diabetes. J Adv Nurs, 2010; 66:1500-1509. doi: 10.1111/j.1365-2648.2010.05329.x. | Excluded because of topic of interest. The main focus was on perceived support from healthcare practitioners and its impact on the motivation of adults with T2D and not maintenance of lifestyle changes over time. |
| Okoro G. Lived experiences of Nigerian immigrants with type 2 diabetes in a large metropolitan area in North Texas. PhD Thesis. Walden University. | Excluded because of topic of interest. The study explored the daily lived experiences of diabetes patients diagnosed with T2D for at least one year. However, it is unclear if participants maintained lifestyle changes over time (>1 yr). |
| Omar H, Busolo D, Hickey J, Gupta N. Health resilience in Arabic-speaking adult refugees with type 2 diabetes: a grounded theory study during the COVID-19 pandemic. Can J Diabet, 2024; 48(2): 82-8. doi: 10.1016/j.jcjd.2023.10.403 | Excluded because of topic of interest. The study explored how adult Arabic-speaking refugees managed their diabetes while resettling during the COVID-19 pandemic. Participants had been diagnosed with T2D for several years, but it is unclear if they maintained lifestyle changes over time (>1 yr). |
| Parry O, Peel E, Douglas M, Lawton J. Issues of cause and control in patient accounts of type 2 diabetes. Health Educ Res, 2006; 21: 97-107. doi: 10.1093/her/cyh044. | Excluded because of topic of interest. The main topic of interest was on T2D patients’ experi-ences and opinions of diabetes services, how these services affect their self-management, not on maintaining lifestyle changes over time. |
| Polonsky WH, Fisher L, Guzman S, Sieber WJ, Philis-Tsimikas A, Edelman SV. Are patients' initial experiences at the diagnosis of type 2 diabetes associated with attitudes and self-management over time? Diabetes Educ, 2010; 36: 828-34. doi: 10.1177/0145721710378539. | Excluded because of type of study design. This was a quantitative study where participants responded to a closed-ended questionnaire. |
| Polzer RL. African Americans and diabetes: spiritual role of the health care provider in self-management. Res Nurs Health, 2007; 30: 164-74. doi: 10.1002/nur.20179. | Excluded because of topic of interest. The main focus was on the spiritual relationship between patients and healthcare providers and its impact on diabetes self-management, not on maintaining lifestyle changes over time. |
| Poon JL, L OH, Kendal H, Sully K, Guy M, Bradley H, et al. Perceptions and experiences of people with obesity and type 2 diabetes around appetite and eating behaviors: a qualitative study. Adv Ther, 2024; 41(5): 2028-49. doi: 10.1007/s12325-024-02846-5 | Excluded because of topic of interest. The study explored eating behaviors and drivers/triggers of food choices in adults with T2D and/or obesity. While some findings concern positive triggers, it is unclear if participants maintained lifestyle changes for over a year. Additionally, findings cannot be separated between those with T2D and those without. |
| Ramirez-Morros A, Berenguera A, Millaruelo L, Buil-Cosiales P, Gomez Garcia C, Cos X, et al. Impact of gender on patient experiences of self-Management in type 2 diabetes: a qualitative study. Patient Prefer Adherence, 2024;18:1885-96. doi: 10.2147/PPA.S466931 | Excluded because of topic of interest. The study explored gender differences in knowledge, attitudes and control and self-management in adult T2D patients. However, the focus of self-management is not maintenance of lifestyle changes over time. |
| Rise MB, Pellerud A, Rygg LO, Steinsbekk A. Making and maintaining lifestyle changes after participating in group based type 2 diabetes self-management educations: a qualitative study. PLOS ONE, 2013; 8: e64009. doi: 10.1371/journal.pone.0064009. | Excluded because of topic of interest.  The main focus was on maintaining lifestyle changes after completion of a three-week group-based education program for diabetes self-management, not on maintaining lifestyle changes over time. |
| Ritholz MD, Beverly EA, Brooks KM, Abrahamson MJ, Weinger K. Barriers and facilitators to self-care communication during medical appointments in the United States for adults with type 2 diabetes. Chronic Illn, 2014; 10: 303–313. doi: 10.1177/1742395314525647. | Excluded because of topic of interest. The main topic of interest was communication barriers and facilitators between patients and healthcare providers regarding diabetes self-care and not maintenance of lifestyle changes over time. |
| Ritonga SH, Decroli E, Prahastuti BS, Usman E, Bachtiar A, Yetti H. Lifestyle of type 2 diabetes mellitus patients with peripheral neuropathy: phenomenological study. J Pakistan Med Assoc, 2024; 74 (Suppl5): S13-S7. DOI: 10.47391/JPMA.Ind-RInC-04 | Excluded because of topic of interest. The study explored lifestyle-related characteristics in people with T2D and peripheral neuropathy. Participants had a mean diabetes duration of six years, but it is unclear if they maintained lifestyle changes over at least a year. The study focuses on behaviour characteristics, not enablers or barriers. |
| Rohloff P. Adults' experiences with type 2 diabetes in rural Guatemala: a qualitative study. J Health Care Poor Underserved, 2023; 34: 208–223. doi: 10.1353/hpu.2023.0014 | Excluded because of topic of interest. The main topic of interest was the experiences and perspectives of adults with T2D regarding the causes, treatments, and curability of the disease and not the maintenance of lifestyle changes over time. |
| Rosenbek Minet LK, Lonvig EM, Henriksen JE, Wagner L. The experience of living with diabetes following a self-management program based on motivational interviewing. Qual Health Res, 2011; 21: 1115-1126. doi: 10.1177/1049732311405066. | Excluded because of type of study participants. The study included both type 1 and type 2 diabetes patients, but it was not possible to separate the findings between the two groups. |
| Roth EG, Girling LM, Chard S, Wallace BH, Eckert JK. Diabetes and the motivated patient: understanding perlocutionary effect in health communication. Health Commun, 2017; 32: 502–508. doi: 10.1080/10410236.2016.1140270 | Excluded because of topic of interest. The main topic interest was the impact of healthcare provider communication on older patients with T2D and not maintenance of lifestyle changes over time. |
| Rutledge S, Hulbert L, Charter-Harris J, Smith A, Owens-Gary M. A qualitative exploration of facilitators and barriers to adopting a healthy lifestyle among Black, Hispanic, and American Indian males with diabetes or at risk for type 2 diabetes. Ethn Health, 2024; 29(4-5): 447-64. doi: 10.1080/13557858.2024.2359377. | Excluded because of topic of interest. The study explores facilitators and barriers to adopting a healthy lifestyle in Black, Hispanic, and American Indian men with diabetes or at risk for T2D. However, the duration of maintained lifestyle changes is unknown, and both participant groups were analyzed together. |
| Savoca M, Miller C. Food selection and eating patterns: themes found among people with type 2 diabetes mellitus. J Nutr Educ, 2001; 33: 224-33. doi: 10.1016/s1499-4046(06)60035-3. | Excluded because of topic of interest. Although the study examines the challenges and strategies related to dietary self-management among adults with T2D, the focus was not on maintenance of lifestyle changes over time. |
| Schoenberg NE, Drungle SC. Barriers to non-insulin dependent diabetes mellitus (NIDDM) self-care practices among older women. J Aging Health, 2001; 13: 443-66. doi: 10.1177/089826430101300401. | Excluded because topic of interest. The main focus was on perceived barriers to self-care among adults with T2B, rather than on barriers encountered when maintaining lifestyle changes over time. |
| Schure M, Goins RT, Jones J, Winchester B, Bradley V. Dietary beliefs and management of older American Indians with type 2 diabetes. J Nutr Educ Behav, 2019; 51: 826-833. doi: 10.1016/j.jneb.2018.11.007. | Excluded because of topic of interest. The main topic was the beliefs, practices, and experiences related to dietary management in adults with T2D and not maintenance of dietary changes over time. |
| Searle A, Ranger E, Zahra J, Tibbitts B, Page A, Cooper A. Engagement in e-cycling and the self-management of type 2 diabetes: a qualitative study in primary care. BJGP Open, 2019;3: bjgpopen18X101638. doi: 10.3399/bjgpopen18X101638. | Excluded because of topic of interest. The main topic of interest was engagement with a 20-week e-cycling intervention and not maintenance of physical activity over time. |
| Seear KH, Lelievre MP, Atkinson DN, Marley JV. 'It's important to make changes': insights about motivators and enablers of healthy lifestyle modification from young Aboriginal men in Western Australia. Int J Environ Res Public Health, 2019; 16: 1063. doi: 10.3390/ijerph16061063 | Excluded because of topic of interest.  Although this study explored motivators and enablers of lifestyle changes in young men with T2D the duration of their maintenance is unclear. |
| Shahabi N, Hosseini Z, Aghamolaei T, Behzad A, Ghanbarnejad A, Dadipoor S. Determinants of adherence to treatment in type 2 diabetic patients: a directed qualitative content analysis based on Pender's health promotion model. Qual Health Res, 2023. doi: 10.1177/10497323231206964. | Excluded because of topic of interest. Although the study explored adherence to treatment (medication, diet, and physical activity) in adults with T2D who had been under treatment for more than 12 months, it is unclear whether participants had maintained lifestyle changes during the same period. |
| Simegn W, Mohammed SA, Moges G. Adherence to self-care practice among type 2 diabetes mellitus patients using the Theory of Planned Behavior and Health Belief Model at comprehensive specialized hospitals of Amhara region, Ethiopia: mixed method. Patient Preference Adher, 2023; 17: 3367-89. | Excluded because of topic of interest. This mixed-methods study assessed adherence to self-care practices among adult T2D patients with at least one year of diagnosis. While many had strategies for self-care, including healthy eating and physical activity, it is unclear if they maintained self-care for over a year. |
| Smith C, McNaughton DA, Meyer S. Client perceptions of group education in the management of type 2 diabetes mellitus in South Australia. Aust J Prim Health, 2015; 22: 360-367. doi: 10.1071/PY15008. | Excluded because of topic of interest. The main topic of interest was to understand perspectives on group education for self-management in adults with T2D and not maintenance of lifestyle changes over time. |
| Spry E, Seear K, Harkin B, O'Donnell V, Maple-Brown L, Atkinson D, et al. Aboriginal young people's experiences of type 2 diabetes diagnosis, management and support: A qualitative study in the Kimberley region of Western Australia. Health Promot J Aust, 2024. doi: 10.1002/hpja.919 | Excluded because of topic of interest. The study explored diabetes experiences and factors impacting self-management in Aboriginal young people aged 12 to 24. It did not focus on maintaining lifestyle changes over time, and results did not distinguish between different age groups. |
| Stubbs M. Empowerment in middle-aged people with diabetes: the importance of working relationships. J Diabetes Nurs, 2007; 11: 190-195. | Excluded because of topic of interest. The main topic was attitudes towards lifestyle changes in T2D patients, how they gained control over their disease, and their perceptions of becoming empowered to manage their disease, rather than maintaining lifestyle changes over time. |
| Tariq O, Rosten C, Huber J. Cultural influences on making nutritional adjustments in type 2 diabetes in Pakistan: the perspectives of people living with diabetes and their family members. Qual Health Res, 2024; 34(6): 562-78. doi: 10.1177/10497323231219392 | Excluded because of topic of interest. The study explored how adults with T2D and their families adjusted their nutrition to health professionals' recommendations, identifying perceived barriers and enablers. Participants had T2D for at least one year, with 13 out of 30 actively making nutritional changes. However, it is unclear if these changes were maintained over at least one year. |
| Thomsen S, Sandbaek A, Agergaard S. Doing physical activity or not: An ethnographic study of the reasoning of healthcare workers and people with type 2 diabetes. Soc Theory Health, 2024. doi: 10.1057/s41285-024-00210-9 | Excluded because of topic of interest. The study explored reasons for engaging in physical activity (PA) or not in people with T2D participating in two municipality healthcare programs involving PA. Participants are likely adults, but their age is not stated. Findings can be separated between those undertaking PA and those not, but it is unclear if PA was maintained for over a year, as the programs lasted only 2-3 months. |
| Tulloch H, Sweet SN, Fortier M, Capstick G, Kenny GP, Sigal RJ. Exercise facilitators and barriers from adoption to maintenance in the diabetes aerobic and resistance exercise trial. Can J Diabetes, 2013; 37: 367-74. doi: 10.1016/j.jcjd.2013.09.002. | Excluded because of topic of interest. The study examined the maintenance of exercise among adults with T2D who participated in an inter-vention. However, maintenance was defined as 9 months rather than 12 months or more. |
| Tuobenyiere J, Mensah GP, Korsah KA. Patient perspective on barriers in type 2 diabetes self-management: a qualitative study. Nurs Open, 2023;10: 7003-7013. doi: 10.1002/nop2.1956. | Excluded because of topic of interest. The main topic of interest was to explore the barriers to self-management among adults with T2D, rather than on barriers encountered when maintaining lifestyle changes over time. |
| Tzeng W-C, Feng H-P. Dietary management in individuals with serious mental illness and comorbid diabetes: a focused ethnography study. J Nurs Res, 2023; 31(5): 1-9. doi: 10.1097/jnr.0000000000000571 | Excluded because of topic of interest. The study explored beliefs and experiences of dietary management in patients with T2D and a serious mental illness. It is unclear how long they had tried to make lifestyle changes. |
| van Smoorenburg AN, Hertroijs DFL, Dekkers T, Elissen AMJ, Melles M. Patients' perspective on self-manage-ment: type 2 diabetes in daily life. BMC Health Serv Res, 2019; 19: 605. doi: 10.1186/s12913-019-4384-7. | Excluded because of topic of interest. The main topic was integration of management into daily routines among adults with T2D without a specific long-term timeframe. |
| Vermeire E, Van Royen P, Coenen S, Wens J, Denekens J. The adherence of type 2 diabetes patients to their therapeutic regimens: a qualitative study from the patient's perspective. Prac Diabetes, 2003; 20: 209-214. doi: 10.1002/pdi.505 | Excluded because of topic of interest. The main topic of interest was health beliefs, communication with caregivers, and problems with adherence to treatment regimens among adults with T2D. The study did not focus on maintenance of lifestyle changes over time. |
| Vilafranca Cartagena M, Arreciado Maranon A, Artigues-Barbera E, Tort-Nasarre G. Successful practices in performing and maintaining physical activity in adults with type 2 diabetes mellitus: A qualitative study. Int J Environ Res Public Health, 2022; 19(21). doi:10.3390/ijerph192114041 | Excluded because of topic of interest. The study explored adults who adhered to physical activity (PA) and who had been diagnosed with T2D for at least two years. However, ee do not know whether they had maintained PA for at least one year. |
| Vicente A, Candila J, Thomas JJ, Gomez Aguilar P, Oliva Aviles C. Living with type 2 diabetes in San Jose Tecoh, Yucatan, Mexico: a phenomenological study. J Transcult Nurs, 2019; 30: 214-221. doi: 10.1177/1043659618790090. | Excluded because of topic of interest. The main topic of interest was to understand the psychosocial aspects and lived experiences of adults with T2D and not maintenance over time. |
| Weiler DM, Crist JD. Diabetes self-management in a latino social environment. Diabetes Educ, 2009; 35: 285-292. doi: 10.1177/0145721708329545. | Excluded because of topic of interest. The main focus was general experiences and transformations that adults undergo after being diagnosed with T2D and not maintenance of lifestyle changes over time. |
| Weller SC, Baer R, Nash A, Perez N. Discovering successful strategies for diabetic self-management: a qualitative comparative study. BMJ Open Diabetes Res Care, 2017; 5: e000349. doi: 10.1136/bmjdrc-2016-000349. | Excluded because of topic of interest. The main focus was to describe self-management practices (e.g. diet, physical activity) in adults with T2D and to identify strategies that distinguish good, fair, and poor glycemic control. However, the maintenance of lifestyle changes over time are not explored. |
| Wilson D, Diji AK-A, Marfo R, Amoh P, Duodu PA, Akyirem S, et al. Dietary adherence among persons with type 2 diabetes: A concurrent mixed methods study. PLOS ONE, 2024; 19(5): e0302914. DOI: 10.1371/journal.pone.0302914. | Excluded because of topic of interest. The study evaluated factors influencing healthier diet adoption via a health app in two T2D patient groups and identified techniques to enhance positive factors and overcome barriers. Despite participants having T2D for over a year, their long-term diet maintenance is unknown |
| Weymiller AJ. Progressive self-curing: a grounded theory study of exercise behavior maintenance in older adults with type 2 diabetes. Phd. Thesis. University of Minnesota Twin Cities. 2009. Available from: <https://hdl.handle.net/11299/58031> | Excluded because of topic of interest. The study focused on exercise maintenance among older adults with type 2 diabetes who were actively exercising. However, it defined maintenance as a period of 6 months or longer rather than 12 months or more. |
| Wong SKW, Soon W, Griva K, Smith HE. Identifying barriers and facilitators to self care in young adults with type 2 diabetes. Diabet Med, 2024; 41(4): e15229. doi: 10.1111/dme.15229 | Excluded because of topic of interest. The study investigated factors influencing self-care behaviors in young adults with T2D, focusing on healthy eating, regular exercise, and medication adherence. However, the duration of their lifestyle changes remains unclear. |
| Zare M, Tarighat-Esfanjani A, Rafraf M, Shaghaghi A, Asghari-Jafarabadi M, Shamshiri M. The barriers and facilitators of self-management among adults with type 2 diabetes mellitus: a Trans-Theoretical Model (TTM)-mased mixed method study in Iran. Diabetes Metab Syndr Obes, 2020; 13: 2687–2699. doi: 10.2147/DMSO.S230083. | Excluded because of topic of interest. The main topic of interest was the stages of change and the factors influencing self-management behaviors among adults with T2B and not maintenance over lifestyle changes over time. |
