# Supplementary material for: What motivates people with type 2 diabetes to maintain lifestyle changes and what challenges do they experience? A qualitative evidence synthesis
Source: PLoS One. 2025 Sep 18;20(9):e0332276. doi: 10.1371/journal.pone.0332276 (PMC12445501; doi:10.1371/journal.pone.0332276)
Supplement: S6 Appendix — (DOCX) [file pone.0332276.s006.docx]

S6 Appendix. Characteristics and methodological limitations of included studies.

| **Hall et al., 2003 [36]** | | | |
| --- | --- | --- | --- |
| **Data extractors:** TB, NO | | **Date of data extraction:** Sept 2022 | **Eligible for inclusion:** Yes |
| **Country:** | | USA, New England | |
| **Participants** | | Number: 5  Age: 55, 60, 62, 65, 82  Gender: 3 males, 2 females  Ethnicity: Caucasus  Socioeconomic status: Middle class | |
| **Methods** | | Study design: Descriptive qualitative study  Data collection methods: In-depth semi structured interviews.  Analytic approach: The data analysis process involved coding the interview transcripts to identify key themes and patterns. Use of thematic analysis approach, reading and re-reading the data, generating codes, and organizing these codes into broader themes. The process was iterative and involved constant comparison of data to refine and validate themes. | |
| **Research question/purpose** | | How to maintain lifestyle changes despite obstacles? Strategies used to overcome the obstacles in relation to diet, exercise and measuring blood sugar are identified | |
| **Result** | | Factors that had a negative impact on diet were hunger pangs, meal planning and little willingness to try new foods. Factors that negatively affected exercise and blood sugar control were physical illness and unplanned/unexpected life events. | |
| **Methodological limitations** | | | |
| **Question** | **Judgement** | **Summary** | |
| **Is the setting and context described sufficiently?** | Yes | Context and setting are described. The study is a descriptive qualitative study conducted in New England USA, describing obstacles that five people with type 2 diabetes experience in everyday life, and strategies they use to maintain lifestyle changes in terms of diet, exercise, and measurement of blood sugar. The description of the study provides an understanding of the geographical setting, the sosio economic status and the timeframe. | |
| **Is the selection strategy described, and is this appropriate?** | Can’t tell | The included participants are described in terms of age, gender, marital status, socioeconomic status, education level and ethnicity. Participants are drawn from a previously unpublished survey of 200 people conducted by the same author. 12 participants were identified as "engaging in behavior change related to diet, exercise, and SMBG for at least a year and reported having maintained this behavior”. 8 individuals were selected because the researcher believed that a small number of individuals would enable the interviews that are necessary "for descriptive data to come to light". The researchers gives partly description of the selection strategy. | |
| **Is the data collection strategy described and justified?** | Yes | The data collection method is well explained. Semi-structured and unstructured face-to-face in-depth interviews with audio recordings and handwritten notes were used. An interview guide with open-ended questions was used as a starting point, and the text describes the process carefully. Interviews were recorded on tape and transcribed. The type of question used was described and applied to all participants. The questions were created with a view to identifying obstacles that arise along the way and strategies to solve this. Attempts were made to avoid leading questions, but follow-up questions were asked to elaborate and clarify the data. Self-reporting may involve people reporting what they "think is correct” | |
| **Is the data analysis described and is it appropriate** | Yes | The method is accounted for. The data analysis was based on the first case and the others were compared to find common features and differences. The authors read the first interview and made one summary to find whether the data were unclear or missing, and the next interview could thus address and elaborate on deficiencies and nuances. To increase the credibility of the interpretations, "two of the participants underwent transcripts of their interviews for accurate interpretations" and "one researcher (nationally known in qualitative research) reviewed the responses from two participants interviews, and this review was then compared with another researcher's interpretation". | |
| **Are the claims/ findings supported by sufficient evidence?** | Yes | Findings described and are based on quotes from various participants. The authors provide details on how many of the participants experienced a certain obstacle or who used a specific strategy to overcome a certain obstacle. | |
| **Are there proof of reflexivity?** | No | There is little or no examples of reflexivity in the text. Interviews could have been supplemented with, for example, diary and observation to elicit a more neutral finding, not just self-reporting. The researcher's own preconceptions and role could have been better expressed. The first interview was read, and a summary was made to find whether the data was unclear or missing. The next interview could thus address, and elaborate on, shortcomings and nuances that the researchers had not thought of. The authors discussed how best to strive for confidence in findings in terms of credibility, fittingness, auditability, and confirmability. During interviews, they thought about the importance of listening, not showing emotions and letting the participants be the expert. Subjects were also asked if they had physical or emotional discomfort that could affect the interview setting. Two participants read the transcribed interview to see that it was correct. One researcher made a summary of two interviews that were compared with the first. There was a lack of description of the researchers’ preconceptions and who they are, what influenced their interest in the study's research questions, their previous experience with qualitative research or with the clinical area being researched (lifestyle change and maintenance in type 2 diabetes patients), possibly preconceptions, etc. They did, however, describe the efforts made during the data collection, such as establishing a relationship of trust with each participant and avoiding communicating anyone’s sense of authority about diabetes | |
| **Does the study show sensitivity to ethical concerns?** | Can`t tell | Prior to the interviews, a summary of the study (informant letter) with a written informed consent was sent to the institutional review board for approval. Upon approval an informant letter was sent to the informants and the informants was called being given the interest behind the study. Unclear ethical approval of the study. | |
| **Other concerns?** | No | Concerns mainly related to selection, lack of evidence of reflexivity and sensitivity to ethical concerns | |
| **Methodological limitations:** | Minor to moderate | | |

| **Peel et al., 2010 [40]** | | | |
| --- | --- | --- | --- |
| **Data extractors:** TB, NO | | **Date of data extraction:** Sept 2022 | **Eligible for inclusion:** Yes |
| **Country** | | Scottland, Lothian region | |
| **Participants** | | Number: 20 participants from general practices and hospitals  Age: 40-80  Gender:11 male, 9 female  Ethnicity: Not specified  Socioeconomic status: Registrar General's classification system: 6(I-II), 5(III N), 8(III M), 1(IV-V) | |
| **Methods** | | Study design: Longitudinal qualitative study.  Data collection methods In-depth interviews over four years (baseline, 6 months, 12 months, 4 years)  Analytic approach**:** The study used a critical realist approach viewing participants' accounts as reflective of their real experiences while considering sociocultural contexts. Interviews were repeatedly read and coded for physical activity content. Data were cross-compared between those who maintained physical activity and those who did not, with sequential analysis conducted to track changes over time | |
| **Research question/purpose** | | Investigate how patients with type 2 implement and maintain physical activity. | |
| **Result** | | Information and advice from health professionals was scarce and not very specific, creating a feeling of lack of interest and lack of support. Only walking as a physical activity seemed to be maintained over time, while a little physical activity decreased in frequency with time. Patients reported that tampering with others, and especially with dogs, helped to maintain activity over time. | |
| **Methodological limitations** | | | |
| **Question** | **Judgement** | **Summary** | |
| **Is the setting and context described sufficiently?** | Yes | Setting and context are accounted for. The study was conducted in Lothian region Scotland and involved participants from 16 general practices and three hospitals. The study aimed to explore the implementation and sustainability of physical activity among patients with type 2 diabetes over four years following their clinical diagnosis. The description of the study provides an understanding of the geographical setting, the healthcare context, and the timeframe. | |
| **Is the selection strategy described, and is this appropriate?** | Yes | The selection process is described and is adequate. The participants were selected from a study in 2002 in which 40 patients were interviewed 3 times over the course of one year (baseline, 6 months, and 12 months) about thoughts, experiences, and adaptations in the post diagnostic period (6 months after diagnosis). The patients have been recruited from both GPs and hospitals.20 people gave a fourth interview 4 years later about how they implement and maintain physical activity. Characteristics of included participants are described in table; and information on age, gender, socioeconomic status, treatment and location is provided. | |
| **Is the data collection strategy described and justified?** | Yes | The data collection strategy is described. Data collection is conducted with face-to-face interviews with question guide available in the article. Notes was written directly after interview by the same interviewer/first author to include context or immediate thoughts/impressions. Interview number four was conducted in the participants' homes, as opposed to the first 3 interviews that were in hospital. The data collection strategy is well-described and justified, aligning with the study's aim to explore the implementation and sustainability of physical activity among patients with type 2 diabetes over an extended period | |
| **Is the data analysis described and is it appropriate** | Yes | Data analysis is well described and appropriate and was performed by reading interviews several times and statements relating to physical activity were coded using NVivo. Notes were read to look for context information. Data were re-read and those who had implemented and maintained physical activity were compared against those who had failed to do so. Data from all four interviews (on the same person) were looked at to discover changes in approach to physical activity over time, considering how and why change has occurred. No information given about how many involved in the analysis. | |
| **Are the claims/ findings supported by sufficient evidence?** | Yes | Findings are described. The findings are based on quotes from various participants. First, general findings are described. From the few who had implemented and maintained physical activity, walking the dog was an important factor and hence 3 main topics. | |
| **Are there proof of reflexivity?** | No | Not described. | |
| **Does the study show sensitivity to ethical concerns?** | Yes | Ethical approval sought to find contact information after 4 years and participants approved audio recordings. All data is marked with pseudonym and location info and name have been changed. | |
| **Other concerns?** | No |  | |
| **Methodological limitations:** | Minor to moderate | | |

| **Wycherley et al., 2011 [45]** | | | |
| --- | --- | --- | --- |
| **Data extractors:** TB, NO | | **Date of data extraction:** Oct 2024 | **Eligible for inclusion:** Yes |
| **Country** | | Australia | |
| **Participants:** | | Number: Thirty participants with T2D  Age: 56,7 ± 7,7  Gender: 22 male, 8 female  Ethnicity: Not specified  Socioeconomic status: Not specified | |
| **Methods** | | Study design: Qualitative study  Data collection methods: Semi structured interviews  Analytic approach: The data analysis process had a content analysis approach with four stages:  1. Initial theme identification by the first author  2. Refinement of themes jointly with two additional authors  3. Manual coding of themes by the first author  4. Joint review and agreement on themes and codes by the three authors. Finally common themes were identified, and the frequency of each theme was tallied and expressed as a percentage of total interviewees. | |
| **Research question/purpose** | | The purpose of the study was to identify factors that enhance or impede the maintenance of healthy lifestyle behaviors in overweight and obese individuals with type 2 diabetes following a 16-week supervised lifestyle intervention program. | |
| **Result** | | Participants retained 8.8 ±8.9 kg of the 13.9 ±6.6 kg weight loss from a supervised lifestyle intervention. Only 23% continued with the full diet program, primarily due to a desire for variety (33%) and larger portion sizes (27%). Those who participated in supervised exercise suggested that access to suitable programs (38), more affordable gym memberships (21%), and personal trainers (17%) would have improved exercise adherence | |
| **Methodological limitations** | | | |
| **Question** | **Judgement** | **Summary** | |
| **Is the setting and context described sufficiently?** | Can`t tell | Context and setting are partly described. The study was conducted as a follow-up to a 16-week research-based supervised lifestyle intervention program. The intervention included a structured energy-restricted diet with or without supervised resistance-exercise training. The initial program was conducted at the Commonwealth Scientific and Industrial Research Organisation (CSIRO) research gymnasium in Adelaide, Australia. The semi-structured interviews were conducted one year after the completion of the 16-week intervention program. | |
| **Is the selection strategy described, and is this appropriate?** | Yes | The selection strategy for the study involved recruiting participants through public advertisements. Eligible participants were overweight or obese, sedentary men and women with type 2 diabetes. Exclusion criteria included known proteinuria, malignancy, a history of liver, kidney, cardiovascular, respiratory, or gastrointestinal disease, uncontrolled hypertension, pregnancy, lactation, smoking, insulin use, musculoskeletal injuries, and recent regular physical exercise. The selection strategy is described and appears appropriate for the study. | |
| **Is the data collection strategy described and justified?** | Yes | The data collection strategy involved conducting semi-structured qualitative interviews with participants who completed the 16-week research-based supervised lifestyle intervention program. The interviews were conducted one year after the commencement of the program. An investigator, who was not part of the original research team, conducted standardized open-ended telephone interviews. The interview questions focused on participants' reasons for participating in the program, difficulties and coping strategies during the program, and factors that impeded or assisted them in maintaining the program components post-intervention. The responses were directly transcribed, and content analyzed for common themes. Semi-structured qualitative interviews allows participants' personal experiences and perceptions and is suitable for understanding behaviors, motivations, and barriers related to maintaining lifestyle changes. The open-ended nature of the interviews provides flexibility for participants to express their thoughts in their own words. Having an external investigator conduct the interviews helps minimize bias and ensures that participants feel comfortable sharing their honest opinions. | |
| **Is the data analysis described and is it appropriate** | Yes | The analysis method is appropriate for qualitative data, as it allows systematic categorization and interpretation of textual data to identify patterns and themes. Multiple authors help ensure the reliability and validity of the findings. Joint refinement and agreement on themes and codes reduce the likelihood of individual bias and enhance the credibility of the analysis. The process followed a four-stage approach: 1. Initial Theme Identification where themes were initially identified by the first author. 2. Refinement: These themes were then refined by the first author and two additional authors. 3. Coding: The initial themes were manually coded by the first author. 4. Review and Agreement: The themes and codes were reviewed and agreed upon by the same three authors. The number of times each theme was mentioned by individual participants was tallied and expressed as a percentage of total interviewees (except where noted). | |
| **Are the claims/ findings supported by sufficient evidence?** | Can`t tell | The claims and findings of the study are supported by both quantitative data (weight loss maintenance) and qualitative data (participant quotes, reported percentages) to support its conclusions. The combination of statistical data and detailed qualitative insights ensures that the findings are well described, but reporting is a bit thin. The results were summarised and described, but there is a lack of support for findings in the form of quotations and all quotations is made by “participant” without any further explanation. | |
| **Are there proof of reflexivity?** | No | The study has some elements of reflexivity, such as using an external interviewer and involving multiple authors in the data analysis to reduce bias, but it does not explicitly address reflexivity and there is little or no examples of reflexivity in the text. The study does not include an explicit statement or discussion about reflexivity. There is no direct reflection on how the researchers' backgrounds, perspectives, or potential biases might have influenced the research process or findings. | |
| **Does the study show sensitivity to ethical concerns?** | Yes | The study demonstrates sensitivity to ethical concerns through informed consent, ethical approval, and the use of an external interviewer to minimize bias and protect participant integrity. The names of the participants have been removed. | |
| **Other concerns?** | No |  | |
| **Methodological**  **limitations:** | Minor to moderate | | |

| **Conlin, 2014 [38]** | | | |
| --- | --- | --- | --- |
| **Data extractors:** TB, NO | | **Date of data extraction:** Sept 2022 | **Eligible for inclusion:** Yes |
| **Country** | | USA | |
| **Participants:** | | Number: 4  Age: Not specified other than > 18  Gender: Male  Ethnicity: Not specified  Socioeconomic status: Not specified | |
| **Methods** | | Study design: Longitudinal qualitative study.  Data collection methods: Semi-structured interviews (three times)  Analytic approach: Line-by-line analysis, theme abstraction, subsumption, contextualization, and numeration. The analysis was iterative and integrative, focusing on the richness and meaning of the data. | |
| **Research question/purpose** | | To examine the experience of sustained successful lifestyle change and achieved long-term positive health outcomes, as well as understanding the factors and conditions that contributed to success in women. | |
| **Result** | | Ten themes were designated as superior to women; 1. Denial, shock and fear 2. Motivated by, but not paralyzed by fear 3. Ownership 4. Lifestyle change incl. approach and routine, regime, self-care, learning 5. Prioritize themselves 6. The system that misses 7. Partnership 8. Good feeling=motivation 9. diabetes-The paradox 10. Being a support for others | |
| **Methodological limitations** | | | |
| **Question** | **Judgement** | **Summary** | |
| **Is the setting and context described sufficiently?** | Yes | Setting and context are described. The study focuses on individuals with type 2 diabetes who have achieved long-term positive health outcomes through sustained lifestyle changes, and it involves detailed interviews to explore their lived experiences, motivations, and challenges. | |
| **Is the selection strategy described, and is this appropriate?** | Yes | Little concern related to the selection of participants. This study was part of a thesis done in collaboration between two researchers. Both researchers followed the same research methodology but collected data from two separate and unique participant groups of four men and four women, respectively. The researchers selected their participants from a group of potential participants who met the study criteria, recruited via doctor referral, social media (LinkedIn) and people referral. | |
| **Is the data collection strategy described and justified?** | Yes | The method chosen is suitable for answering the research question and the data collection is well described. Individual in-depth interviews with semi-structured open-ended questions and follow-up questions that went into specific topics or led to new topics. Three interviews of approximately 60 minutes each were conducted on each of the participants for 1-3 weeks. Interview 1 dealt with the identification of factors that may have led to the diabetes diagnosis, the experience of having a diagnosis, the acute reaction to having received a diagnosis. Interview 2 allowed the participants to reconstruct the successful experience with type 2 diabetes in the context in which this occurred. Interview 3 allowed participants to reflect on the experience and its significance. | |
| **Is the data analysis described and is it appropriate** | Yes | The analysis method seems appropriate. Data were analyzed by following the IPA structure involving line-by-line analysis, identification of themes within individual cases and later across individual cases, interpretation, development of structure illustrating relationships, organization and analysis, development of history and interpretation, reflection. | |
| **Are the claims/ findings supported by sufficient evidence?** | Yes | Evidence exists for statements and conclusions. Claims and findings appear to be accounted for or substantiated. Themes and sub-themes have been created that are supported by quotes. There are no statements or conclusions that have not been accounted for or justified. | |
| **Are there proof of reflexivity?** | Yes | There are examples of reflexivity. One's own preconceptions and role are described. Interviews were adjusted along the way. Statements from participants were validated during the interviews by repeating and adjusting questions. Researcher's own perceptions, beliefs and processes is described. | |
| **Does the study show sensitivity to ethical concerns?** | Yes | Principles of ethical research practice were followed. The participants signed written consent prior to the interview, which also included permission to record and transcribe. Oral and written information to ensure participants understood? Interview questions are attached, and the researchers’ pilot-tested the questions prior to the interviews. The researchers followed the participants' pace and direction in the interviews. | |
| **Other concerns?** | No |  | |
| **Methodological**  **limitations:** | Minor | | |

| **Phelps, 2014 [39]** | | | |
| --- | --- | --- | --- |
| **Data extractors:** TB, NO | | **Date of data extraction:** Sept 2022 | **Eligible for inclusion:** Yes |
| **Country** | | USA | |
| **Participants** | | Number: 4  Age: Not specified other than > 18  Gender: Female  Ethnicity: Not specified  Socioeconomic status: Not specified | |
| **Methods** | | Study design: Longitudinal qualitative study.  Data collection methods: Semi-structured interviews (three times)  Analytic approach**:** Line-by-line analysis, theme abstraction, subsumption, contextualization, and numeration. The analysis was iterative and integrative, focusing on the richness and meaning of the data. | |
| **Research question/purpose** | | To seek the experience of sustained successful lifestyle change, and achieved long-term positive health outcomes, as well as understanding motivational factors and conditions that contributed to success in men. | |
| **Result** | | Seven overarching themes of importance to men:1: Hendings as triggers 2: Ownership of the disease 3: Motivation 4: Self-care 5: The developed self  6: The diabetes effect 7: Support that matters | |
| **Methodological limitations** | | | |
| **Question** | **Judgement** | **Summary** | |
| **Is the setting and context described sufficiently?** | Yes | Setting and context are described. The study focuses on individuals with type 2 diabetes who have achieved long-term positive health outcomes through sustained lifestyle changes, and it involves detailed interviews to explore their lived experiences, motivations, and challenges. | |
| **Is the selection strategy described, and is this appropriate?** | Yes | Little concern related to the selection of participants. This study was part of a thesis done in collaboration between two researchers. Both researchers followed the same research methodology but collected data from two separate and unique participant groups of four men and four women, respectively. The researchers selected their participants from a group of potential participants who met the study criteria, recruited via doctor referral, social media (LinkedIn) and people referral. | |
| **Is the data collection strategy described and justified?** | Yes | The method chosen is suitable for answering the research question and the data collection is well described. Individual in-depth interviews with semi-structured open-ended questions and follow-up questions that went into specific topics or led to new topics. Three interviews of approximately 60 minutes each were conducted on each of the participants for 1-3 weeks. Interview 1 dealt with the identification of factors that may have led to the diabetes diagnosis, the experience of having a diagnosis, the acute reaction to having received a diagnosis. Interview 2 allowed the participants to reconstruct the successful experience with type 2 diabetes in the context in which this occurred. Interview 3 allowed participants to reflect on the experience and its significance. | |
| **Is the data analysis described and is it appropriate** | Yes | The analysis method seems appropriate. Data were analyzed by following the IPA structure involving line-by-line analysis, identification of themes within individual cases and later across individual cases, interpretation, development of structure illustrating relationships, organization and analysis, development of history and interpretation, reflection. | |
| **Are the claims/ findings supported by sufficient evidence?** | Yes | Evidence exists for statements and conclusions. Claims and findings appear to be accounted for or substantiated. Themes and sub-themes have been created that are supported by quotes. There are no statements or conclusions that have not been accounted for or justified. | |
| **Are there proof of reflexivity?** | Yes | There are examples of reflexivity. One's own preconceptions and role are described. Interviews were adjusted along the way. Statements from participants were validated during the interviews by repeating and adjusting questions. Researcher's own perceptions, beliefs and processes is described. | |
| **Does the study show sensitivity to ethical concerns?** | Yes | Principles of ethical research practice were followed. The participants signed written consent prior to the interview, which also included permission to record and transcribe. Oral and written information to ensure participants understood? Interview questions are attached, and the researchers’ pilot-tested the questions prior to the interviews. The researchers followed the participants' pace and direction in the interviews. | |
| **Other concerns?** | No |  | |
| **Methodological**  **limitations:** | Minor | | |

| **Walker et al., 2018 [41]** | | | |
| --- | --- | --- | --- |
| **Data extractors:** TB, NO | | **Date of data extraction:** Sept 2022 | **Eligible for inclusion:** Yes |
| **Country** | | Denmark, Copenhagen | |
| **Participants:** | | Number: 6 from the InterWalk trial.  Age: 41, 57, 57, 66, 70  Gender: 5 males, one female  Ethnicity: Not specified  Socioeconomic status: Not specified | |
| **Methods** | | **Study design:** Longitudinal qualitative study.  **Data collection methods** Semi-structured interviews conducted initiation, 12 weeks and 52 weeks post-enrollment  **Analytic approach:** Systematic Text Condensation (STC) with guidance from Self-Determination Theory (SDT) | |
| **Research question/purpose** | | Examine motivating factors for implementing and maintaining physical activity after completing the rehabilitation program. | |
| **Result** | | Successful behavioural changes were linked to a willingness to create new structure in everyday life in order to create more independence. The feeling of having the opportunity to participate in physical activity was driven by knowledge, practical experiences and progression. Feeling stagnant weakened motivation. The feeling of having fun was crucial for exercising physical activity over a long period of time. | |
| **Methodological limitations** | | | |
| **Question** | **Judgement** | **Summary** | |
| **Is the setting and context described sufficiently?** | Yes | Setting and context are accounted for. Lifestyle changes including physical activity have shown beneficial health effects, but adherence to lifestyle changes has been reported to vary greatly. The study was conducted within the InterWalk trial, a rehabilitation program for patients with Type 2 Diabetes (T2D) in Copenhagen, involving interval walking training facilitated by a smartphone app. Participants were interviewed at three different points: at the initiation of the program, after 12-week completion, and 52 weeks after enrollment. The interviews were conducted either at the research unit or the health promotion center. | |
| **Is the selection strategy described, and is this appropriate?** | Yes | The selection process works thoroughly and is clearly presented. Participants were recruited from the InterWalk study, a placebo-controlled randomised trial with three arms in which the control group was randomised to follow the standard rehabilitation program with education and exercise facilitated by the municipality. The other two groups were both given training programs via an app to conduct interval-scheduled walking training. Detailed descriptions of the InterWalk study are referred to in the text. Strategic selection is used. Participants from different places, of different ages and with different genders are included, in addition, the researchers have focused on retrieving participants both early and late during the study. A table with participant characteristics can be found in the study. | |
| **Is the data collection strategy described and justified?** | Yes | The choice of method is justified and relevant. The data collection is well described. Individual semi-structured interviews were conducted at three different times by the first author; one week after inclusion, after the end of the rehabilitation program, and a year after inclusion. The interviews were conducted in one quiet room in the research department or at the health center. The interview guide contained topics related to initiation, implementation and maintenance of physical activity and was prepared by two of the authors based on their own reflections, as well as literature on the topic. The interview guide was adjusted between interviews to include topics that emerge. Statements were validated by repetitive questions and cross-interview validation. Audio recordings of interviews and choice of method are justified.  Not discussed data saturation. | |
| **Is the data analysis described and is it appropriate** | Yes | The data analysis has been accounted for and seems appropriate. All interviews were conducted and transcribed by the first author. Pauses in language, laughter, and similar ways of expressing oneself were noted to ensure correct perception of the situation. Malterud's systematic text analysis (STC) was used in analysis. The first part was inductive with a full-text review where potential topics were considered. In the second part, information was identified on motivating factors for physical activity and this information was then coded in different groups where each group represented different aspects of motivation. The next step in the analysis had a deductive approach with an interpretive focus. Main groups with headings were created, before the third phase of coding and division of subgroups was started. The fourth step was to create an analytical text of the codes that was re-contextualized and assessed in the context of the entire material to ensure validity. The codes were constantly reflected on, challenged, and documented. | |
| **Are the claims/ findings supported by sufficient evidence?** | Yes | There are no statements or conclusions that have not been accounted for or lack justification. Themes and sub-themes have been created that are supported by quotes from the interviewees. | |
| **Are there proof of reflexivity?** | Can’t tell | There are examples of reflexivity in the research process, but somewhat inadequate discussion of one's own preconceptions and role. The interview guide was adjusted along the way to investigate and validate emerging topics. Statements from participants were validated during the interviews by repeating questions and comparing them with other interviews. The researchers have not discussed their own role, in terms of influence in formulating research questions, data collection, selection and setting. | |
| **Does the study show sensitivity to ethical concerns?** | Yes | There are few or no concerns about ethical principles. Ethical principles were safeguarded by providing the participants with oral and written information about the purpose of the study and having at least 24 hours to assess participation. Consent forms were used for interviews and audio recordings. The interviews were conducted in a separate room. The study was reported to the Regional Committee for Medical and Health Research Ethics. | |
| **Other concerns?** | No |  | |
| **Methodological**  **limitations:** | Minor | | |

| **Schmidt et al., 2020 [42]** | | | |
| --- | --- | --- | --- |
| **Data extractors:** TB, NO | | **Date of data extraction:** Sept 2022 | **Eligible for inclusion:** Yes |
| **Country** | | Denmark | |
| **Participants:** | | Number: 6 patients from the U-TURN trial.  Age: 41, 49, 50, 56, 69, 74  Gender: 3 male, 3 female  Ethnicity: Not specified  Socioeconomic status: Not specified | |
| **Methods** | | Study design: Longitudinal qualitative study.  Data collection methods In-depth interviews conducted 12- and 18-months post-intervention  Analytic approach: Systematic text condensation with an inductive approach | |
| **Research question/purpose** | | Investigate and identify factors that influence motivation and barriers to creating and maintaining lifestyle changes after participation in a lifestyle change intervention. | |
| **Result** | | Social support, the ability to relate to behavioral change, as well as support from health professionals, all contributed to the maintenance of results. Being able to identify with and accept a new lifestyle was also related to achieving results over time. To maintain results over a longer period, patients had to adopt and accept that the new lifestyle became part of their social life. | |
| **Methodological limitations** | | | |
| **Question** | **Judgement** | **Summary** | |
| **Is the setting and context described sufficiently?** | Yes | Setting and context are linked to other content and are clearly described. The study was conducted within the context of the U-TURN trial, a 12-month intensive lifestyle intervention for patients with Type 2 Diabetes (T2D). Participants underwent structured exercise, dietary counseling, and additional lifestyle modifications. Six participants from the intervention group were selected for in-depth interviews conducted 12- and 18-months post-intervention. The study setting included participant homes or research offices, providing a comprehensive understanding of their experiences and challenges in maintaining lifestyle changes | |
| **Is the selection strategy described, and is this appropriate?** | Yes | No concerns about the selection of participants. Participants for this study were recruited from the intervention group in the U-TURN randomised controlled trial (RCT). All patients from the intervention group were informed and given the opportunity to participate in interviews. Selection criteria included gender representation, diversity in residential areas, and different exercise groups to ensure diversity and data saturation. This study has a comprehensive and representative sample of participants. Table with characteristics of participants is included in the study. | |
| **Is the data collection strategy described and justified?** | Yes | The method chosen is suitable for answering the research question and the data collection is well described. Individual in-depth interviews were conducted after 12 months to investigate the participants' behavioral change and motivation, and after 18 months to identify barriers and facilitate maintenance of lifestyle changes. The interviews were conducted in the participants' homes or in a separate room in the research office based on the participants' own wishes. All interviews were conducted by the first author, and an observer also had the opportunity to ask questions and participate in reflections on each of the interviews. The method is justified, and the interview guide was discussed with the co-author. The interview guide was adjusted along the way and explained how and why. Data saturation is discussed. | |
| **Is the data analysis described and is it appropriate** | Yes | The data analysis is described, and appropriate and in-depth interviews are well suited for this type of study where the purpose is to explore experiences and perceptions. The data is collected over time and can say something about retention over time. | |
| **Are the claims/ findings supported by sufficient evidence?** | Yes | There are no statements or conclusions that have not been accounted for or justified. Themes and sub-themes have been created that are supported by quotes. The codes from the analysis were continuously discussed and disagreements discussed with a third person. | |
| **Are there proof of reflexivity?** | Can’t tell | There are examples of reflexivity in the research process. The researchers acknowledge the presence of an observer during the interviews to ensure validity and provide critical reflections on the interview technique. The first author conducted small analyses between the interviews, reflecting on data and adjusting the interview guide. The duration of the interviews was 60–120 minutes. Follow-up questions were carefully prepared, field notes on the environment and participants' behavior, actions, and emotions were obtained after each interview. Researchers have not discussed their own role and potential bias and impact in terms of research questions and data collection. | |
| **Does the study show sensitivity to ethical concerns?** | Yes | Principles for good ethical research practice were followed. The participants received oral and written information about the purpose of the study. Consent forms were obtained, and the participants consented to audio recordings. The participants were reminded of volunteerism and the possibility of resigning. The names of the participants have been replaced by gender and age. According to Danish legislation does not require qualitative studies approval from an ethics committee. | |
| **Other concerns?** | No |  | |
| **Methodological**  **limitations:** | Minor | | |

| **Janssen et al., 2023 [47]** | | | |
| --- | --- | --- | --- |
| **Data extractors:** TB, NO | | **Date of data extraction:** Feb 2025 | **Eligible for inclusion:** Yes |
| **Country** | | Canada | |
| **Participants:** | | Number:12  Age: 54-81 a clinical diagnosis of T2D  Gender: 7 female, 5 male  Ethnicity: not specified  Socioeconomic status: Education level: 5 university, 4 high school, 3 college.  Employment: 8 retired, 4 working | |
| **Methods** | | Study design: A mixed methods case series design.  Data collection methods: purposive sampling, follow-up telephone interviews were conducted at one, six, and 12-months.  Analytic approach: thematic analysis was employed to analyze interviews | |
| **Research question/purpose** | | The purpose of the study was to:  1) measure change in fasting blood glucose, blood pressure, anthropometrics), and physical function after completing an eight-week education and exercise program for adults with T2D; and  2) explore the experience of exercise continuation in people living with T2D at one-year follow-up. | |
| **Result** | | Clinically significant improvements were observed for waist circumference, systolic blood pressure, six-minute walk test (6MWT), timed up- and-go test (TUG), 30-second chair stand test (CST) and arm curls. Three themes emerged from interviews that described participant reflections and experiences with a supervised education and exercise program for management of their T2D: 1) medical management; 2) lifestyle management; and 3) finding what works. | |
| **Methodological limitations** | | | |
| **Question** | Judgement | Summary | |
| **Is the setting and context described sufficiently?** | Can’t tell | The study take place in a midsized city in Ontario, Canada. The GFAL-D, an eight-week education and exercise program, was provided free of charge to study participants at a local center for older adults during 2018–2019. The GFAL-D program was led by a physiotherapy student, certified as both a GFAL-D and Senior Fitness Instructor, working with a licensed physiotherapist. | |
| **Is the selection strategy described, and is this appropriate?** | Yes | Participants were recruited using flyers posted at medical offices and community centers offering programs and social events for adults. The participants included were ≥ 18 years, had a clinical diagnosis of T2D (glycated hemoglobin (A1C) ≥ 6.5%), owned a smartphone or tablet, had cognitive and physical ability to participate in an education and exercise program, and were fluent in English. | |
| **Is the data collection strategy described and justified?** | Yes | Participants completed two one-hour exercise sessions and one one-hour education session per week for eight weeks. Blood glucose, blood pressure, body mass index (BMI), waist circumference, and physical function were measured at baseline and after completing the program. Semi-structured individual interviews adopting a qualitative descriptive methodology were conducted by telephone one-, six-, and 12-months post-GFAL-D Eleven of the 12 participants completed all three interviews which were on average 45 minutes (range: 25–55 minutes). Examples of interview questions. | |
| **Is the data analysis described and is it appropriate** | Yes | BREQ-2 questionnaires were scored and graphed using Microsoft Excel 365.Fasting blood glucose, blood pressure, anthropometrics, and physical function measures were summarized and presented in a separate scenario for each individual participant. Rather than calculating statistical significance, which is not the purpose of a case series design, outcome measures were compared to the minimal clinically important difference (MCID) for each outcome. Audio recorded interviews were transcribed and thematic analysis was employed by the authors based on the method of Braun and Clarke. The researchers embodied a pragmatist, considering multiple perspectives from participants and positions from both qualitative and quantitative data, making pragmatism a suitable paradigm for mixed methods reses. The authors analysing the interviews together. Interviews completed across three time points of the one-year study were analyzed as a group, from which three themes emerged. Data analysis and collection were ongoing and iterative. Codes and themes were discussed between the authors, and consensus was achieved on developed themes. Figure shows the three main themes and codes generated by the researchers. | |
| **Are the claims/ findings supported by sufficient evidence?** | Yes | Theme 1: Medical management. Theme 2: Lifestyle management. Theme 3: Finding what works.  There are no statements or conclusions that have not been accounted for or lack justification. Themes have been created that are supported by quotes from the interviews. | |
| **Are there proof of reflexivity?** | No | Not described | |
| **Does the study show sensitivity to ethical concerns?** | Can’t tell | This study was approved by Western University’s Health Sciences Research Ethics Board and written informed consent were obtained from all participants. Interviews were recorded following expression of informed consent by participants | |
| **Other concerns?** | No |  | |
| **Methodological**  **limitations:** | Minor | | |

| **Ribu et al., 2024 [44]** | | | |
| --- | --- | --- | --- |
| **Data extractors:** TB, NO | | **Date of data extraction:** Feb 2025 | **Eligible for inclusion:** Yes |
| **Country** | | Norway | |
| **Participants:** | | Number:26  Age: aged ≥18 years  Gender: Not specified  Ethnicity: Not specified  Socioeconomic status: Not specified | |
| **Methods** | | Study design: grounded theory approach with a constant comparative method  Data collection methods: face‐to‐face, open‐ended, in‐depth interviews in the participant's home, the interviewer's office or by telephone  Analytic approach: The data analysis for this study was conducted in accordance with grounded theory (GT) methodology, aligning data analysis with ongoing data collection | |
| **Research question/purpose** | | The aim of this study was to attain in‐depth knowledge of the persons' perceptions and responses to what was happening in their lives and to identify the conditions and social patterns that affect self‐management. Research questions were: What are the conditions to which patients with type 2 diabetes perceive within the process of their illness and to which they respond? What are their responses (actions–interactions or strategies) to the identified conditions? What are the outcomes of their responses? How do patients perceive their situation? | |
| **Result** | | The struggle to self‐manage and maintain new habits can be more or less difficult depending on the patient's perceived conditions. We identified three situations illustrated in a diagram: one where there is less struggle to let go of old habits, a second where there is more of a struggle to balance between what individuals want to do and what they ought to do and a third where they are giving up struggling. Study findings show that healthcare personnel must consciously seek to understand how patients perceive their own situation. | |
| **Methodological limitations** | | | |
| **Question** | Judgement | Summary | |
| **Is the setting and context described sufficiently?** | Yes | Setting and context of the study are described sufficiently. Participants were recruited from the two intervention groups in the Norwegian three‐armed RCT of the EU project RENEWING HEALTH (RH). Adults with type 2 diabetes aged ≥18 years, with HbA1c level ≥ 7.1% and those who were able to complete questionnaires in Norwegian. Interviews done in the participant's home, the interviewer's office or by telephone for those who lived far away. | |
| **Is the selection strategy described, and is this appropriate?** | Yes | No concerns about the selection of participants. The selection strategy is described and appropriate. The study included participants who had consented to follow-up interviews at the outset of their enrollment. After completing a year in the original RCT, these participants were assessed for eligibility, and interviews were conducted upon their exit from the study until theoretical saturation was achieved. Out of the initial 50 eligible participants, 15 could not participate due to various reasons such as poor health or being unreachable. From the remaining 35, seven declined to be interviewed, and two became too ill. Ultimately, 26 participants were interviewed. | |
| **Is the data collection strategy described and justified?** | Yes | The study involved conducting 26 face-to-face, open-ended, in-depth interviews, which took place either in the participant's home, the interviewer's office, or via telephone for those living far away. Although all interviews were audiotaped, only 24 were analysed due to technical issues with the recording device. The transcriptions were produced verbatim and assessed by two of the authors. The duration of the interviews varied from 22 to 90 minutes, with an average length of 48 minutes. Each participant participated in one interview, and memos were written post-interview to inform future discussions. The subsequent interviews were designed to build on insights gained from previous ones. The research team also revisited the data for additional clarification and insights throughout the analysis. A semi-structured interview guide was employed during the interview process. | |
| **Is the data analysis described and is it appropriate** | Yes | The data analysis for this study was conducted in accordance with grounded theory (GT) methodology, aligning data analysis with ongoing data collection. This involved writing memos that summarized and integrated various categories around a core concept to develop a theoretical framework.  The initial analysis was grounded in open coding, where the focus was on incidents rather than individuals. The concept of "struggling" emerged as a predominant theme, reflecting their ongoing efforts, setbacks, rationalizations, and re-tries. This led to the identification of a core category: "struggling between wanting to do something and feeling obligated to do it."  The second phase of the analysis employed axial coding, utilizing Corbin and Strauss's analytic paradigm to explore potential connections among concepts. This paradigm includes aspects such as conditions, action–interactions, and consequences.  In the results section, the research presents contexts that may clarify the action–interactions against specific conditions and expected consequences, shedding light on why certain individuals experience greater struggles than others. Through this systematic linking of concepts, the groundwork for developing a theory based on Corbin's theoretical coding framework was laid out.. | |
| **Are the claims/ findings supported by sufficient evidence?** | Yes | Themes and sub-themes have been created and are supported with examples. A Figure has been created to show the struggling between “ought to do” and “want to do”. | |
| **Are there proof of reflexivity?** | No | There are few or no examples of reflexivity in the text. There is a lack of description of the researcher’s biases and who they are, what influenced their interest in the research question, their previous experience conducting qualitative research or the clinical field being researched. | |
| **Does the study show sensitivity to ethical concerns?** | Yes | The study was approved by the Regional Committee for Medical and Health Research Ethics in Norway (REC no 2010/427). Participants were provided with both written and verbal information during the larger Randomized Controlled Trial (RCT), including guarantees of full confidentiality and the right to withdraw from the study at any point without needing to provide a reason. Participants also consented to being contacted for follow-up interviews after the RCT. Upon being contacted for the interview, they received comprehensive written and verbal information regarding the interview process, as well as general procedural details. Following each interview, the data was anonymized by substituting names with codes, and the audio recordings were securely stored in a locked safe. | |
| **Other concerns?** | No |  | |
| **Methodological**  **limitations:** | Minor | | |

| **Van den Burg et al., 2024** **[43]** | | | |
| --- | --- | --- | --- |
| **Data extractors:** TB, NO | | **Date of data extraction:** Feb 2025 | **Eligible for inclusion:** Yes |
| **Country** | | Netherlands | |
| **Participants:** | | Number: 100 recruited, 92 completed the assessments  Age: 18 – 75 years  Gender: 48 men and 43 women  Ethnicity: Not specified  Socioeconomic status: In the FMD group: 40.8% had low, 26.5% intermediate, 28.6% high education, and 4.1% were unknown. In the control group: 34.9% had low, 30.2% intermediate, and 34.9% high education levels. | |
| **Methods** | | Study design: mixed method  Data collection methods: Qualitative data collected through focus group discussions with participants in the FMD group to explore their experiences with self-initiated lifestyle changes and to identify barriers and facilitators of these changes.  Analytic approach: Theoretical Domains Framework to analyze focus group data, identifying barriers and facilitators related to lifestyle changes and participant experiences during the FMD program. | |
| **Research question/purpose** | | The primary research question or purpose of the study was to investigate whether following a fasting-mimicking diet (FMD) program could influence lifestyle behaviors in patients with type 2 diabetes, specifically focusing on changes in diet quality and physical activity. The study aimed to evaluate if the periodic use of an FMD would encourage self-initiated lifestyle changes beyond the structured intervention period and identify any barriers or facilitators to such changes. | |
| **Result** | | The study concluded that while the FMD did not significantly change diet quality in the quantitative analysis, it did increase physical activity levels and fostered an environment for self-initiated lifestyle improvements through qualitative findings. | |
| **Methodological limitations** | | | |
| **Question** | Judgement | Summary | |
| **Is the setting and context described sufficiently?** | Yes | The setting and the study are described sufficiently. The study was conducted in the Netherlands at the Leiden University Medical Center. Participants were individuals with type 2 diabetes managed in primary care settings. They followed a fasting-mimicking diet for five consecutive days each month over 12 months. The study used a mix of quantitative questionnaires and qualitative focus groups to assess lifestyle changes and experiences. | |
| **Is the selection strategy described, and is this appropriate?** | Yes | Participants were selected from general practices in the Netherlands using computerized record searches, reviewed by GPs to ensure eligibility. This approach was appropriate, targeting individuals with type 2 diabetes managed in primary care settings, aligned with the study's aims. The selection strategy for the qualitative part is described. Participants from the FMD group who completed the follow-up were purposively sampled to ensure diversity in gender, age, BMI, and adherence to the diet. This approach was appropriate to capture a wide range of experiences and perspectives. | |
| **Is the data collection strategy described and justified?** | Yes | The data collection used validated questionnaires for diet and physical activity and focus groups for in-depth participant experiences. This mix provided both quantitative and qualitative insights, effectively addressing the study's objectives. | |
| **Is the data analysis described and is it appropriate** | Yes | Yes, the data analysis is described and is appropriate. Quantitative Analysis: Utilized linear mixed models to assess changes over time in diet quality and physical activity. This method is suitable for handling repeated measures data and accounting for intra-participant variability. Qualitative Analysis: Employed the Theoretical Domains Framework to analyze focus group data, identifying barriers and facilitators to lifestyle changes. This framework is appropriate for studying behavioral change processes. | |
| **Are the claims/ findings supported by sufficient evidence?** | Yes | The study's findings are well-supported by the use of mixed models for quantitative data and thematic analysis of qualitative focus group discussions, providing contextual insights into participants' experiences. This combination offers robust evidence. | |
| **Are there proof of reflexivity?** | No | There are few or no examples of reflexivity in the text. | |
| **Does the study show sensitivity to ethical concerns?** | Yes | The study demonstrates ethical sensitivity through approval by the Medical Research Ethics Committee and obtaining informed consent from participants. Additionally, measures were taken to ensure participant privacy by anonymizing data, reflecting a commitment to ethical research practices. | |
| **Other concerns?** | No |  | |
| **Methodological limitations:** | Minor | | |

| **Muchiri et al., 2024** **[48]** | | | |
| --- | --- | --- | --- |
| **Data extractors:** TB, NO | | **Date of data extraction:** Feb 2025 | **Eligible for inclusion:** Yes |
| **Country** | | South Africa | |
| **Participants:** | | Number: 24 in focus group intervention group, 19 in control group  Age: 18 – 75 years  Gender: 8 men and 35 women  Ethnicity: Not specified  Socioeconomic status: Participants were primarily from uninsured and low-income backgrounds. The participants had at least high school education. | |
| **Methods** | | Study design: Phenomenological study design.  Data collection methods: Qualitative data collected through focus-group discussions (4-6 participants) in intervention group and individual interviews with control group.  Analytic approach: Thematic framework. This approach involved familiarization, generating a thematic framework, coding, and mapping/interpreting the data to identify key issues and meanings. | |
| **Research question/purpose** | | The research aimed to investigate how a randomized controlled trial of an adapted diabetes nutrition education program was received by adults with sub-optimally controlled type 2 diabetes, and to understand the factors influencing the program's outcomes and participant retention. | |
| **Result** | | Participants expressed high satisfaction with the diabetes nutrition education program and found the educational materials, especially the poster, valuable for both themselves and their families. They reported benefits such as improved diabetes knowledge, dietary self-care skills, better health, and increased motivation for self-care, which encouraged them to complete the program. Some participants noted positive dietary and physical activity behavior changes. Despite positive feedback, the program had limited clinical impact on HbA1c levels and faced a high attrition rate, with approximately 62% retention at 12 months. Participants suggested enhancements such as more medical content and including family members in some sessions for greater support. | |
| **Methodological limitations** | | | |
| **Question** | Judgement | Summary | |
| **Is the setting and context described sufficiently?** | Yes | The setting and context are described sufficiently. The study was conducted in a diabetes outpatient clinic at a public tertiary teaching hospital in Pretoria, South Africa, primarily serving uninsured and low-income patients. Participants were adults with sub-optimally controlled type 2 diabetes (HbA1c ≥8%), and the context involved adapting a diabetes nutrition education program to fit the needs of a tertiary healthcare setting. | |
| **Is the selection strategy described, and is this appropriate?** | Yes | The selection strategy is described and appears appropriate. Participants were adults aged 40-70 years, with type 2 diabetes for at least one year and sub-optimal diabetes control (HbA1c ≥8%). They were recruited from a diabetes outpatient clinic in Pretoria, South Africa, and required the ability to understand English. This targeted selection ensured the inclusion of individuals who could benefit most from the nutrition education program | |
| **Is the data collection strategy described and justified?** | Yes | The data collection strategy is described and justified. The study used focus-group discussions (FGDs) and individual interviews (II) to gather qualitative data. FGDs were conducted at two points: after the curriculum completion and at the study's end, allowing for exploration of participants' experiences over time. Individual interviews with control group participants provided additional insights. This strategy helped capture detailed, personal experiences and perceptions of the program. | |
| **Is the data analysis described and is it appropriate** | Yes | The data analysis is described and appropriate. The study employed framework analysis, which involved familiarization with the data, developing a thematic framework, coding, and interpreting key themes. This method was suitable for exploring both predefined and emerging categories, allowing an in-depth understanding of participants' experiences and perceptions of the nutrition education program. | |
| **Are the claims/ findings supported by sufficient evidence?** | Yes | The claims and findings are supported by sufficient evidence. The study provides quotes from participants that illustrate the perceived benefits and challenges of the nutrition education program. These quotes and thematic findings align well with the reported satisfaction, behavioral changes, and program impact, giving a comprehensive view of participants' experiences. | |
| **Are there proof of reflexivity?** | Can`t tell | There are few or no examples of reflexivity in the text although the researchers engaged in regular debriefing sessions and discussions among the moderation team to reflect on the data collection and analysis. They also had their analysis reviewed by a peer experienced in qualitative research who was not involved in the study, ensuring objectivity and consideration of different perspectives. | |
| **Does the study show sensitivity to ethical concerns?** | Yes | Ethical approval was obtained from the Research Ethics Committee, Faculty of Health Sciences at the University of Pretoria. Participants were informed about the study and their involvement was voluntary, adhering to ethical standards for research involving human subjects. | |
| **Other concerns?** | No |  | |
| **Methodological limitations:** | Minor | | |

| **Campbell et al. 2024 [46]** | | | |
| --- | --- | --- | --- |
| **Data extractors:** TB, NO | | **Date of data extraction:** Des 2024 | **Eligible for inclusion:** Yes |
| **Country** | | New Zealand | |
| **Participants:** | | Number: 40 participants (12 participants with T2D interviewed at 12 months)  Age: 20 - 65  Gender: 8 female, 4 male  Ethnicity: The participants were mainly of Māori (54%) or Pacific (24%) ethnicity.  Socioeconomic status: Not specified | |
| **Methods** | | Study design: Qualitative study within a RCT.  Data collection methods: Semi structured interviews conducted after 3- and 12 months  Analytic approach: Inductive thematic analysis with a six phrase process. | |
| **Research question/purpose** | | Explore the experiences and acceptability of the Diabetes Remission Clinical Trial (DiRECT) intervention among a predominantly Māori and Pacific Island population living with type 2 diabetes or prediabetes in Aotearoa New Zealand. The study aimed to understand participants' perspectives on the DiRECT intervention, particularly considering the strong cultural emphases on food and shared eating, and to evaluate the acceptability of the intervention in this diverse population. | |
| **Result** | | The study found that the DiRECT intervention was acceptable for weight loss and maintenance in a predominantly Māori and Pacific Island population with type 2 diabetes or prediabetes in New Zealand. Key results include the importance of culturally relevant and tailored support in lifestyle interventions. | |
| **Methodological limitations** | | | |
| **Question** | **Judgement** | **Summary** | |
| **Is the setting and context described sufficiently?** | Yes | Setting and context of the study are described sufficiently. The study was conducted in Aotearoa New Zealand, specifically within a Māori primary care provider. It involved participants from predominantly Māori and Pacific Island communities (strong cultural emphasis on food and shared eating practices). The study's context is detailed by its focus on understanding the acceptability and experiences of the DiRECT intervention among these populations. | |
| **Is the selection strategy described, and is this appropriate?** | Yes | No concerns about the selection of participants. The selection strategy is described and appropriate. Participants were aged 20–65 years with type 2 diabetes or prediabetes, obesity, and a desire to lose weight. Exclusions included recent heart issues, insulin use, and pregnancy. The strategy focused on Māori and Pacific Island communities to explore cultural influences on the intervention's acceptability. | |
| **Is the data collection strategy described and justified?** | Yes | Semi-structured interviews were conducted at 3 and 12 months to capture participants' perspectives and experiences of the DiRECT intervention. This approach allowed individual experiences and provided flexibility for participants to discuss topics relevant to them. Interviews at two different times helped capture changes and developments in participants'. The use of semi-structured interviews is justified in qualitative studies to understand the experiences. | |
| **Is the data analysis described and is it appropriate** | Yes | Reflexive thematic analysis was used, involving six phases: familiarization, coding, generating themes, reviewing, refining, and writing up findings. This method suits the qualitative study's aim to explore participants' experiences and identify meaningful patterns in their responses. | |
| **Are the claims/ findings supported by sufficient evidence?** | Yes | The qualitative analysis is based on semi-structured interviews with participants, capturing perspectives and experiences. The findings are illustrated with direct quotes from participants. The findings were discussed with additional researchers to enhance the analysis. Coded transcripts were reviewed by another researcher to provide an alternative perspective and strengthen the coding framework. Additionally, two researchers with expertise in kaupapa Māori and one in Pacific culture gave feedback on aspects of the analysis relevant to culture and cultural identity. | |
| **Are there proof of reflexivity?** | Yes | The researchers acknowledged their own backgrounds, biases, and assumptions, and how these could influence the research process. Reflexive thematic analysis was used, which inherently involves reflecting on how the researchers' perspectives might affect the interpretation of the data. The analysis was conducted with an awareness of the researchers' positions and included feedback from researchers with expertise in kaupapa Māori and Pacific culture to ensure cultural sensitivity and appropriateness in the interpretation of the findings. | |
| **Does the study show sensitivity to ethical concerns?** | Yes | Ethical approval was obtained from the relevant ethics committee, and the trial was prospectively registered, ensuring adherence to ethical research standards. Participants provided informed written consent before enrollment, and they were free to withdraw from the study at any time without disadvantage. The study emphasized participant-driven research, conducted in response to patients' requests for weight loss support, and all aspects of the study design, analysis, and reporting were informed by a multicultural research team. | |
| **Other concerns?** | No |  | |
| **Methodological**  **limitations:** | Minor | | |
