# Supplementary material for: What motivates people with type 2 diabetes to maintain lifestyle changes and what challenges do they experience? A qualitative evidence synthesis
Source: PLoS One. 2025 Sep 18;20(9):e0332276. doi: 10.1371/journal.pone.0332276 (PMC12445501; doi:10.1371/journal.pone.0332276)
Supplement: S7 Appendix — (DOCX) [file pone.0332276.s007.docx]

S7 Appendix. Summary of qualitative findings table.

| **What motivates people with type two diabetes to maintain lifestyle changes and what challenges do they experience?** | | | |
| --- | --- | --- | --- |
| **Summary of review finding** | **Studies contributing to the review finding** | **GRADE‐CERQual assessment of confidence in the evidence** | **Explanation of GRADE‐CERQual assessment** |
| **What motivates the maintaining of lifestyle change?** | | | |
| **Finding 1.** **People with type 2 diabetes found it helpful to get support from others when trying to maintain lifestyle changes. “Having someone” around who acted supportively was reported to create commitment and the desire to succeed. This support could come from close relationships, colleagues, exercise groups, healthcare professionals or someone in the same situation. People also perceived tailored support from healthcare professionals as positive for maintaining changes in lifestyle.** | Hall et al., 2003 [36]; Wycherley et al., 2011 [45]; Conlin, 2014 [38]; Phelps, 2014 [39]; Ribu et al., 2019 [44]; Schmidt et al., 2020 [42]; Campbell et al., 2024 [46]; Muchiri et al. 2024 [48]. | High confidence | Based on minor concerns related to methodological limitations and no or very minor concerns about adequacy, relevance, and coherence. |
| **Finding 2. People with type 2 diabetes found that choosing activities they enjoyed and that fitted into their personal circumstances promoted maintenance of lifestyle changes. Walking as an activity was experienced as simple and with a high degree of feasibility that could be implemented in daily life through for example exercise, housework, gardening or walking the dog.** | Hall et al., 2003 [36]; Peel et al., 2010 [40]; Wycherley et al., 2011 [45]; Conlin, 2014 [38]; Phelps, 2014 [39]; Walker et al., 2018 [41]; Janssen et al., 2023 [47]; Van den Burg et al., 2024 [43]. | Moderate confidence | Based on minor concerns related to methodological limitations and relevance. No or very minor concerns about the coherence and adequacy. |
| **Finding 3**. **People with type 2 diabetes saw the company of others when carrying out physical activity, such as joining an exercise group or going for a walk with others, as positive for maintaining lifestyle changes and found it motivating to have regular appointments with or obligations towards others.** | Peel et al., 2010 [40]; Wycherley et al., 2011 [45]; Walker et al., 2018 [41]; Schmidt et al., 2020 [42]; Van den Burg et al., 2024 [43]; Muchiri et al. 2024 [48]. | Moderate confidence | Based on minor concerns related to methodological limitations, relevance, and adequacy. No or very minor concerns about coherence. |
| **Finding 4.** **People with type 2 diabetes described how seeing results from their lifestyle changes had a positive effect on their motivation. This included achievements such as weight loss.** | Hall et al., 2003 [36]; Wycherley et al., 2011 [45 Conlin, 2014 [38]; Phelps, 2014 [39]; Walker et al., 2018 [41]; Schmidt et al., 2020 [42]; Campbell et al., 2024 [46]; Van den Burg et al., 2024 [43]; Muchiri et al. 2024 [48]. | High confidence | Based on minor concerns related to methodological limitations. There are no or very minor concerns about coherence, relevance, and adequacy. |
| **Finding 5. People with type 2 diabetes viewed increased knowledge about the disease as a source of motivation for maintaining their lifestyle changes. Sharing this knowledge with others with the same illness could also strengthen people’s commitment to maintain their own lifestyle changes.** | Hall et al., 2003 [36]; Conlin, 2014 [38]; Phelps, 2014 [39]; Muchiri et al. 2024 [48]; Van den Burg et al., 2024 [43]. | Moderate confidence | Based on minor concerns related to methodological limitations and adequacy. There are no or very minor concerns about coherence and relevance. |
| **Finding 6. People with type 2 diabetes described how a fear of the complications of diabetes (for instance, a fear of nursing homes, sickness and, death) motivated them to change their lifestyles and maintain these changes over time.** | Hall et al., 2003 [36]; Conlin, 2014 [38]; Phelps, 2014 [40]; Walker et al., 2018 [41]; Schmidt et al., 2020 [42]. | Moderate confidence | Based on minor concerns related to methodological limitations and relevance. There are no or very minor concerns about coherence and adequacy. |
| **Finding 7. People with type 2 diabetes believed that taking control of the disease could help them maintain lifestyle changes. This involved accepting the disease and its consequences, prioritizing oneself, setting goals, and having strategies and structure in everyday life. Some felt that personal qualities such as being competitive, goal-oriented, good at planning, autonomous, mentally strong, self-aware and optimistic were important for taking control and being able to maintain lifestyle change.** | Hall et al., 2003 [36]; Wycherley et al., 2011 [45]; Conlin, 2014 [38]; Phelps, 2014 [39]; Walker et al., 2018 [41]; Ribu et al., 2019 [44]; Schmidt et al., 2020 [42]; Janssen et al., 2023 [47]; Campbell et al., 2024 [46]; Muchiri et al. 2024 [48]. | High confidence | Based on minor concerns related to methodological limitations. There are no or very minor concerns about coherence, relevance and adequacy. |
| **What challenges the maintenance of lifestyle change?** | | | |
| **Finding 8. People with type 2 diabetes described several factors that negatively affected their motivation to maintain exercise over time. These included a lack of progress, unachieved goals and limited understanding of the benefits of physical activity, in addition to a complicated relationship with physical activity from the past, feeling uncomfortable in gyms, the experience of exercise as time-consuming and little enjoyment of the activity.** | Peel et al., 2010 [40]; Wycherley et al., 2011 [45]; Conlin, 2014 [38]; Phelps, 2014 [39]; Walker et al., 2018 [41]; Ribu et al., 2019 [44]; Schmidt et al., 2020 [42]. | Moderate confidence | Based on minor concerns related to methodological limitations and relevance. There are no or very minor concerns about coherence and adequacy. |
| **Finding 9.** **People with type 2 diabetes described how their own physical limitations and illness, in addition to practical and social conditions, made it challenging to exercise regularly.** | Hall et al., 2003 [36]; Peel et al., 2010 [40]; Wycherley et al., 2011 [45]; Phelps, 2014 [39]; Walker et al., 2018 [41]; Schmidt et al., 2020 [42]. | Moderate confidence | Based on minor concerns related to methodological limitations and relevance. There are no or very minor concerns about coherence and adequacy. |
| **Finding 10. People with type 2 diabetes found that lifestyle changes tied to diet and exercise limited their flexibility. The desire to regain a sense of autonomy and freedom of choice in everyday life created challenges, and it was difficult to see the new lifestyle as "one's own"** | Hall et al., 2003 [36]; Wycherley et al., 2011 [45]; Schmidt et al., 2020 [42]; Campbell et al., 2024 [46]. | Low confidence | Based on minor concerns related to methodological limitations and relevance. There are no or very minor concerns about coherence and moderate concerns about adequacy. |
| **Finding 11. People with type 2 diabetes experienced that healthcare professionals lacked knowledge about and interest in the disease and additional lifestyle changes.** | Peel et al., 2010 [40]; Conlin, 2014 [38]; Phelps, 2014 [39]; Schmidt et al., 2020 [42]. | Moderate confidence | Based on minor concerns related to methodological limitations and relevance. There are no or very minor concerns about coherence and adequacy. |
| **Finding 12. People with type 2 diabetes described a lack of regular check-ups and follow-up from healthcare professionals and linked this to a feeling of not being important enough, which had an impact on their maintenance of lifestyle changes.** | Phelps, 2014 [39]; Ribu et al., 2019 [44]; Schmidt et al., 2020 [42]; Van den Burg et al., 2024 [43]; Campbell et al., 2024 [46]. | Moderate confidence | Based on minor concerns related to methodological limitations, relevance and adequacy. There are no or very minor concerns about coherence. |
| **Finding 13. People with type 2 diabetes found it challenging to take the disease seriously due to few symptoms. Some described that they didn`t take responsibility for their own health, which made it difficult to maintain lifestyle changes.** | Conlin, 2014 [38]; Phelps, 2014 [39]; Schmidt et al., 2020 [42]. | Low confidence | Based on minor concerns related to methodological limitations. Moderate concerns related to relevance and adequacy. There are no or very minor concerns about coherence. |
