# Supplementary material for: What motivates people with type 2 diabetes to maintain lifestyle changes and what challenges do they experience? A qualitative evidence synthesis
Source: PLoS One. 2025 Sep 18;20(9):e0332276. doi: 10.1371/journal.pone.0332276 (PMC12445501; doi:10.1371/journal.pone.0332276)
Supplement: S8 Appendix — (DOCX) [file pone.0332276.s008.docx]

| **Finding 1. People with type 2 diabetes found it helpful to get support from others when trying to maintain lifestyle changes. “Having someone” around who acted supportively was reported to create commitment and the desire to succeed. This support could come from close relationships, colleagues, exercise groups, healthcare professionals or someone in the same situation. People also perceived tailored support from healthcare professionals as positive for maintaining changes in lifestyle.** | |
| --- | --- |
| **Assessment for each GRADE-CERQual component** | |
| *Methodological limitations* | Minor concerns based on assessments of the studies where two studies had minor to moderate limitations and six studies had minor methodological limitations.  Concerns are mainly related to insufficient justification for the selection of participants, lack of evidence for reflexivity and sensitivity to ethical concerns in one study. It is not considered that the concerns had significance for the finding. |
| *Coherence* | No or very minor concerns. |
| *Relevance* | No or very minor concerns. All the studies described people above the age of 18 with type 2 diabetes and their experiences of what motivates them to maintain lifestyle changes for 12 months or more. Although most of the studies except one (South Africa) were from Western countries (USA, Scotland, Denmark, Norway, Australia, New Zealand), it was not considered to be a concern of significant importance for the finding. |
| *Adequacy* | No or very minor concerns. |
| **Overall GRADE-CERQual assessment and explanation** | |
| High confidence | Based on minor concerns related to methodological limitations and no or very minor concerns about adequacy, relevance and coherence. |
| **Contributing studies** | |
| Hall et al., 2003 [36]; Wycherley et al., 2011 [45]; Conlin, 2014 [38]; Phelps, 2014 [39]; Ribu et al., 2019 [44]; Schmidt et al., 2020 [42]; Campbell et al., 2024 [46]; Muchiri et al. 2024 [48]. | |

S8 Appendix. Evidence profiles.

| **Finding 2. People with type 2 diabetes found that choosing activities they enjoyed and that fitted into their personal circumstances promoted maintenance of lifestyle changes. Walking as an activity was experienced as simple and with a high degree of feasibility that could be implemented in daily life through for example exercise, housework, gardening or walking the dog.** | |
| --- | --- |
| **Assessment for each GRADE-CERQual component** | |
| *Methodological limitations* | Minor concerns based on assessments of the studies where three studies had minor to moderate limitations and five studies had minor methodological limitations. Concerns were mainly related to insufficient justification for the selection of participants, lack of evidence for reflexivity and sensitivity to ethical concerns. |
| *Coherence* | No or very minor concerns. |
| *Relevance* | Minor concerns because all the studies described people above 18 years of age with type two diabetes and their experiences with what motivated them to maintain lifestyle changes after 12 months. Downgraded because the studies only were from high-income countries (Europe, USA, Australia, Canada) and walking is not necessarily an activity that is feasible in all cultures/countries. |
| *Adequacy* | No or very minor concerns. Few participants in all the studies, but rich data from several participants as two of the studies only deal with the maintenance of physical activity. |
| **Overall GRADE-CERQual assessment and explanation** | |
| Moderate confidence | Based on minor concerns related to methodological limitations and relevance. No or very minor concerns about the coherence and adequacy. |
| **Contributing studies** | |
| Hall et al., 2003 [36]; Peel et al., 2010 [40]; Wycherley et al., 2011 [45]; Conlin, 2014 [38]; Phelps, 2014 [39];  Walker et al., 2018 [41]; Janssen et al., 2023 [47]; Van den Burg et al., 2024 [43]. | |

| **Finding 3. People with type 2 diabetes saw the company of others when carrying out physical activity, such as joining an exercise group or going for a walk with others, as positive for maintaining lifestyle changes and found it motivating to have regular appointments with or obligations towards others.** | |
| --- | --- |
| **Assessment for each GRADE-CERQual component** | |
| *Methodological limitations* | Minor concerns based on assessments of the studies where two studies had minor to moderate limitations and four studies had minor methodological limitations.  Concerns mainly related to lack of evidence in reflexivity and sensitivity to ethical concerns. |
| *Coherence* | No or very minor concerns. |
| *Relevance* | Minor concerns because all the studies described people over 18 years of age with type two diabetes and their experiences with what motivates them to maintain lifestyle changes after 12 months. Downgraded because the studies only were from high-income countries (Europe, Canada, Australia) and being in an exercise group/going for walks is not necessarily an activity that is carried out in all cultures/countries) |
| *Adequacy* | No or very minor concerns. |
| **Overall GRADE-CERQual assessment and explanation** | |
| Moderate confidence | Based on minor concerns related to methodological limitations, relevance and adequacy. No or very minor concerns about coherence. |
| **Contributing studies** | |
| Peel et al., 2010 [40]; Wycherley et al., 2011 [45]; Walker et al., 2018 [41]; Schmidt et al., 2020 [42]; Van den Burg et al., 2024 [43]; Muchiri et al. 2024 [48]. | |

| **Finding 4. People with type 2 diabetes described how seeing results from their lifestyle changes had a positive effect on their motivation. This included achievements such as weight loss.** | |
| --- | --- |
| **Assessment for each GRADE-CERQual component** | |
| *Methodological limitations* | Minor concerns based on assessments of the studies where two studies had minor to moderate limitations and seven studies had minor methodological limitations. Concerns were mainly related to insufficient justification for the selection of participants, lack of evidence for reflexivity and sensitivity to ethical concerns. |
| *Coherence* | No or very minor concerns. |
| *Relevance* | No or very minor concerns. All the studies described people above the age of 18 with type 2 diabetes and their experiences of what motivates them to maintain lifestyle changes for 12 months or more. Although most of the studies were from Western countries (USA, Scotland, Denmark, Netherlands, Australia, New Zealand) except one (South Africa), it was not considered to be a concern of significant importance for the finding. |
| *Adequacy* | No or very minor concerns. |
| **Overall GRADE-CERQual assessment and explanation** | |
| High confidence | Based on minor concerns related to methodological limitations. There are no or very minor concerns about coherence, relevance and adequacy. |
| **Contributing studies** | |
| Hall et al., 2003 [36]; Wycherley et al., 2011 [45]; Conlin, 2014 [38]; Phelps, 2014 [39]; Walker et al., 2018 [41]; Schmidt et al., 2020 [42]; Campbell et al., 2024 [46]; Van den Burg et al., 2024 [43]; Muchiri et al. 2024 [48]. | |

| **Finding 5. People with type 2 diabetes viewed increased knowledge about the disease as a source of motivation for maintaining their lifestyle changes. Sharing this knowledge with others with the same illness could also strengthen people’s commitment to maintain their own lifestyle changes.** | |
| --- | --- |
| **Assessment for each GRADE-CERQual component** | |
| *Methodological limitations* | Minor concerns based on assessments of the studies where one study had minor to moderate limitations and four studies had minor methodological limitations. Concerns were mainly related to insufficient justification for the selection of participants, lack of evidence for reflexivity and sensitivity to ethical concerns. |
| *Coherence* | No or very minor concerns. |
| *Relevance* | No or very minor concerns. All the studies described people above the age of 18 with type 2 diabetes and their experiences of what motivates them to maintain lifestyle changes for 12 months or more. Although most of the studies were from high income countries (USA, Netherlands) except one (South Africa), it was not considered to be a concern of significant importance for the finding. |
| *Adequacy* | Minor concerns. The underlying data was relatively thin. |
| **Overall GRADE-CERQual assessment and explanation** | |
| Moderate confidence | Based on minor concerns related to methodological limitations and adequacy. There are no or very minor concerns about coherence and relevance. |
| **Contributing studies** | |
| Hall et al., 2003 [36]; Conlin, 2014 [38]; Phelps, 2014 [39]; Muchiri et al. 2024 [48]; Van den Burg et al., 2024 [43]. | |

| **Finding 6. People with type 2 diabetes described how a fear of the complications of diabetes (for instance, a fear of nursing homes, sickness and, death) motivated them to change their lifestyles and maintain these changes over time.** | |
| --- | --- |
| **Assessment for each GRADE-CERQual component** | |
| *Methodological limitations* | Minor concerns based on assessments of the studies where one study had minor to moderate limitations and four studies had minor methodological limitations. Concerns were mainly related to insufficient justification for the selection of participants, lack of evidence for reflexivity and sensitivity to ethical concerns. |
| *Coherence* | No or very minor concerns. |
| *Relevance* | Minor concerns. All the studies described people above the age of 18 with type 2 diabetes and their experiences of what motivates them to maintain lifestyle changes for 12 months or more. Although all of the studies were only from Denmark and USA, it was not considered to be a concern of significant importance for the finding. |
| *Adequacy* | No or very minor concerns. |
| **Overall GRADE-CERQual assessment and explanation** | |
| Moderate confidence | Based on minor concerns related to methodological limitations and relevance. There are no or very minor concerns about coherence and adequacy. |
| **Contributing studies** | |
| Hall et al., 2003 [36]; Conlin, 2014 [38]; Phelps, 2014 [39]; Walker et al., 2018 [41]; Schmidt et al., 2020 [42]. | |

| **Finding 7. People with type 2 diabetes believed that taking control of the disease could help them maintain lifestyle changes. This involved accepting the disease and its consequences, prioritizing oneself, setting goals, and having strategies and structure in everyday life. Some felt that personal qualities such as being competitive, goal-oriented, good at planning, autonomous, mentally strong, self-aware and optimistic were important for taking control and being able to maintain lifestyle change.** | |
| --- | --- |
| **Assessment for each GRADE-CERQual component** | |
| *Methodological limitations* | Minor concerns based on assessments of the studies where two studies had minor to moderate limitations and eight studies had minor methodological limitations. Concerns were mainly related to insufficient justification for the selection of participants, lack of evidence for reflexivity and sensitivity to ethical concerns. |
| *Coherence* | No or very minor concerns. |
| *Relevance* | No or very minor concerns. All the studies described people above the age of 18 with type 2 diabetes and their experiences of what motivates them to maintain lifestyle changes for 12 months or more. Although most of the studies were only from high income countries (Norway, Denmark, USA, Canada, Australia and New Zealand) except one (South Africa), it was not considered to be a concern of significant importance for the finding. |
| *Adequacy* | No or very minor concerns. |
| **Overall GRADE-CERQual assessment and explanation** | |
| High confidence | Based on minor concerns related to methodological limitations. There are no or very minor concerns about coherence, relevance and adequacy. |
| **Contributing studies** | |
| Hall et al., 2003 [36]; Wycherley et al., 2011 [45]; Conlin, 2014 [38]; Phelps, 2014 [38]; Walker et al., 2018 [41]; Ribu et al., 2019 [44]; Schmidt et al., 2020 [42]; Janssen et al., 2023 [47]; Campbell et al., 2024 [46]; Muchiri et al. 2024 [48]. | |

| **Finding 8. People with type 2 diabetes described several factors that negatively affected their motivation to maintain exercise over time. These included a lack of progress, unachieved goals and limited understanding of the benefits of physical activity, in addition to a complicated relationship with physical activity from the past, feeling uncomfortable in gyms, the experience of exercise as time-consuming and little enjoyment of the activity.** | |
| --- | --- |
| **Assessment for each GRADE-CERQual component** | |
| *Methodological limitations* | Minor concerns based on assessments of the studies where two studies had minor to moderate limitations and five studies had minor methodological limitations. Concerns were mainly related to insufficient justification for the selection of participants, lack of evidence for reflexivity and sensitivity to ethical concerns. |
| *Coherence* | No or very minor concerns. |
| *Relevance* | Minor concerns. All the studies described people above the age of 18 with type 2 diabetes and their experiences of what motivates them to maintain lifestyle changes for 12 months or more. The studies came from high-income settings, and it is possible that this setting differs from other countries/settings. |
| *Adequacy* | No or very minor concerns. |
| **Overall GRADE-CERQual assessment and explanation** | |
| Moderate confidence | Based on minor concerns related to methodological limitations and relevance. There are no or very minor concerns about coherence and adequacy. |
| **Contributing studies** | |
| Peel et al., 2010 [40]; Wycherley et al., 2011 [45]; Conlin, 2014 [38]; Phelps, 2014 [39]; Walker et al., 2018 [41]; Ribu et al., 2019 [44]; Schmidt et al., 2020 [42]. | |

| **Finding 9. People with type 2 diabetes described how their own physical limitations and illness, in addition to practical and social conditions, made it challenging to exercise regularly.** | |
| --- | --- |
| **Assessment for each GRADE-CERQual component** | |
| *Methodological limitations* | Minor concerns based on assessments of the studies where three studies had minor to moderate limitations and three studies had minor methodological limitations. Concerns were mainly related to insufficient justification for the selection of participants, lack of evidence for reflexivity and sensitivity to ethical concerns. |
| *Coherence* | No or very minor concerns. |
| *Relevance* | Minor concerns. All the studies described people above the age of 18 with type 2 diabetes and their experiences of what motivates them to maintain lifestyle changes for 12 months or more. The studies came from high-income settings, and it is possible that this setting differs from other countries/settings. |
| *Adequacy* | No or very minor concerns. |
| **Overall GRADE-CERQual assessment and explanation** | |
| Moderate confidence | Based on minor concerns related to methodological limitations and relevance. There are no or very minor concerns about coherence and adequacy. |
| **Contributing studies** | |
| Hall et al., 2003 [36]; Peel et al., 2010 [40]; Wycherley et al., 2011 [45]; Phelps, 2014 [38]; Walker et al., 2018 [41]; Schmidt et al., 2020 [42]. | |

| **Finding 10.** **People with type 2 diabetes found that lifestyle changes tied to diet and exercise limited their flexibility.** **The desire to regain a sense of autonomy and freedom of choice in everyday life created challenges, and it was difficult to see the new lifestyle as "one's own"** | |
| --- | --- |
| **Assessment for each GRADE-CERQual component** | |
| *Methodological limitations* | Minor concerns based on assessments of the studies where two studies had minor to moderate limitations and two studies had minor methodological limitations. Concerns were mainly related to insufficient justification for the selection of participants, lack of evidence for reflexivity and sensitivity to ethical concerns. |
| *Coherence* | No or very minor concerns. |
| *Relevance* | Minor concerns. All the studies described people above the age of 18 with type 2 diabetes and their experiences of what motivates them to maintain lifestyle changes for 12 months or more. The studies came from high-income settings, and it is possible that this setting differs from other countries/settings. |
| *Adequacy* | Moderate concerns regarding few studies and thin data. |
| **Overall GRADE-CERQual assessment and explanation** | |
| Low confidence | Based on minor concerns related to methodological limitations and relevance. There are no or very minor concerns about coherence and moderate concerns about adequacy. |
| **Contributing studies** | |
| Hall et al., 2003 [36]; Wycherley et al., 2011 [45]; Schmidt et al., 2020 [42]; Campbell et al., 2024 [46]. | |

| **Finding 11. People with type 2 diabetes experienced that healthcare professionals lacked knowledge about and interest in the disease and additional lifestyle changes.** | |
| --- | --- |
| **Assessment for each GRADE-CERQual component** | |
| *Methodological limitations* | Minor concerns based on assessments of the studies where one study had minor to moderate and three studies had minor methodological limitations. Concerns were mainly related to insufficient justification for the selection of participants, lack of evidence for reflexivity and sensitivity to ethical concerns. |
| *Coherence* | No or very minor concerns. |
| *Relevance* | Minor concerns. All the studies described people above the age of 18 with type 2 diabetes and their experiences of what motivates them to maintain lifestyle changes for 12 months or more. The studies came from high-income settings, and it is possible that this setting differs from other countries/settings, especially regarding different health systems. |
| *Adequacy* | No or very minor concerns. |
| **Overall GRADE-CERQual assessment and explanation** | |
| Moderate confidence | Based on minor concerns related to methodological limitations and relevance. There are no or very minor concerns about coherence and adequacy. |
| **Contributing studies** | |
| Peel et al., 2010 [40]; Conlin, 2014 [38]; Phelps, 2014 [39]; Schmidt et al., 2020 [42]. | |

| **Finding 12. People with type 2 diabetes described a lack of regular check-ups and follow-up from healthcare professionals and linked this to a feeling of not being important enough, which had an impact on their maintenance of lifestyle changes.** | |
| --- | --- |
| **Assessment for each GRADE-CERQual component** | |
| *Methodological limitations* | Minor concerns based on assessments of the studies where all four studies had minor methodological limitations. Concerns were mainly related to insufficient justification for the selection of participants, lack of evidence for reflexivity and sensitivity to ethical concerns. |
| *Coherence* | No or very minor concerns. |
| *Relevance* | Minor concerns. All the studies described people above the age of 18 with type 2 diabetes and their experiences of what motivates them to maintain lifestyle changes for 12 months or more. The studies came from high-income settings and it is possible that this setting differs from other countries/settings, especially regarding different health systems. |
| *Adequacy* | Minor concerns regarding relative few studies and thin data. |
| **Overall GRADE-CERQual assessment and explanation** | |
| Moderate confidence | Based on minor concerns related to methodological limitations, relevance and adequacy. There are no or very minor concerns about coherence. |
| **Contributing studies** | |
| Phelps, 2014 [39]; Ribu et al., 2019 [44]; Schmidt et al., 2020 [42]; Van den Burg et al., 2024 [43]; Campbell et al., 2024 [46]. | |

| **Finding 13. People with type 2 diabetes found it challenging to take the disease seriously due to few symptoms. Some described that they didn`t take responsibility for their own health, which made it difficult to maintain lifestyle changes.** | |
| --- | --- |
| **Assessment for each GRADE-CERQual component** | |
| *Methodological limitations* | Minor concerns based on assessments of the studies where both studies had minor methodological limitations. Concerns were mainly related to insufficient justification for the selection of participants, lack of evidence for reflexivity and sensitivity to ethical concerns. |
| *Coherence* | No or very minor concerns. |
| *Relevance* | Moderate concerns. All the studies described people above the age of 18 with type 2 diabetes and their experiences of what motivates them to maintain lifestyle changes for 12 months or more. The studies came from high-income settings, and it is possible that this setting differs from other countries/settings. Lacking data about the participants background, knowledge, and views. |
| *Adequacy* | Moderate concerns regarding few studies and thin data. |
| **Overall GRADE-CERQual assessment and explanation** | |
| Low confidence | Based on minor concerns related to methodological limitations. Moderate concerns related to relevance and adequacy. There are no or very minor concerns about coherence. |
| **Contributing studies** | |
| Conlin, 2014 [38]; Phelps, 2014 [39]; Schmidt et al., 2020 [42]. | |
