# Supplementary material for: What motivates people with type 2 diabetes to maintain lifestyle changes and what challenges do they experience? A qualitative evidence synthesis
Source: PLoS One. 2025 Sep 18;20(9):e0332276. doi: 10.1371/journal.pone.0332276 (PMC12445501; doi:10.1371/journal.pone.0332276)
Supplement: Attachment — Submitted filename: PLOS ONE comments.docx [file pone.0332276.s009.docx]

Manuscript Number: PONE-D-24-00773

Full Title: What motivates people with type 2 diabetes to maintain lifestyle changes and what challenges do they experience? A qualitative evidence synthesis.

Reviewer Comments

I thank the editor for the opportunity given to review this interesting paper.

The authors have taken an effort to review significant papers to conclude it to practical questions needed in the treatment strategy of type -2 diabetes.

Authors claim that this paper is the first of its kind in T2DM management by patients. However, there are such papers already published earlier (doi: [10.1186/1472-6963-14-348](https://doi.org/10.1186%2F1472-6963-14-348)).

This paper is a motivated work of the authors to work on an evidence synthesis on T2DM. However, the authors have not included the iterations conducted to reach only six studies, even though there was no restrictions in the range of time limits and languages of the study.

There was no mention about the understanding of the languages of the study published other than English.

There was also no mention about the data management during the process of evidence synthesis.

**Recommendation**

With this minor revisions, the manuscript can be considered for further process of acceptance for the journal.
