# Supplementary material for: What motivates people with type 2 diabetes to maintain lifestyle changes and what challenges do they experience? A qualitative evidence synthesis
Source: PLoS One. 2025 Sep 18;20(9):e0332276. doi: 10.1371/journal.pone.0332276 (PMC12445501; doi:10.1371/journal.pone.0332276)
Supplement: Attachment — Submitted filename: Response to reviewers.docx [file pone.0332276.s011.docx]

**PONE-D-24-00773**

**What motivates people with type 2 diabetes to maintain lifestyle changes and what challenges do they experience? A qualitative evidence synthesis.**

Thank you for giving us the opportunity to submit a revised manuscript. We appreciate the feedback on our paper and are grateful for the opportunity to improve through your comments. Please see our response to the reviewers’ comments and concerns below.

| **Reviewers’ comments** | **Authors’ response** | **Changes made to the manuscript** |
| --- | --- | --- |
| **Reviewer #1:**  I thank the editor for the opportunity given to review this interesting paper. The authors have taken an effort to review significant papers to conclude it to practical questions needed in the treatment strategy of type -2 diabetes.  Authors claim that this paper is the first of its kind in T2DM management by patients. However, there are such papers already published earlier (doi: 10.1186/1472-6963-14-348). | Thank you for pointing this out, and reviewer #1 is correct about a similar published review. However the review only included studies from the UK and the review authors did not specify whether the included studies focused on maintaining lifestyle changes for a minimum of 12 months. The review focused on how people with type 2 diabetes see self-management strategies as helpful or not, not on maintaining lifestyle changes for at least 12 months.  We also checked the included qualitative studies in the UK review (n=12) and tested them against our inclusion criteria. One of the studies was included in our review (Peel et al, 2010); the other studies were excluded because of topic of interest (as they did not focus on maintenance of lifestyle changes for 12 months or more). | We have referenced the review in the introduction of our QES (line 83). |
| **Reviewer #1:**  This paper is a motivated work of the authors to work on an evidence synthesis on T2DM. However, the authors have not included the iterations conducted to reach only six studies, even though there was no restrictions in the range of time limits and languages of the study. | We understand the reviewers concerns about the few studies included in the review. The previous version of the manuscript did not clearly indicate that many studies identified during the initial screening (title/abstract) potentially met our inclusion criteria but were excluded during the full-text screening. Additionally, we did not elaborate on the reasons for exclusion either in the manuscript text or in a separate appendix. The primary reason for excluding studies was that they did not focus on maintaining lifestyle changes for 12 months or longer (i.e. the topic of interest in the studies did not comply with the topic of interest in our inclusion criteria).  While compiling the list of reasons for excluding studies from our review, we realized that one study (Wycherley et al., 2011, ref #44) excluded from the synthesis should have been included. The findings from this study are now integrated into the review. We also discovered that one of the included studies (Hall et al., 2003) was reported in two publications (scientific article and a thesis, respectively). Both publications are integrated into the review.  Finally, we decided to update our literature search, as the previous search was conducted a year ago at the time of revision. This update led to the inclusion of five additional studies, bringing the total number of studies in the review to twelve. Moreover, our assessments of confidence in the evidence supporting the findings was changed for the following findings:   - Finding 5. Acquiring knowledge: from ‘low’ to ‘moderate’. - Finding 7. Taking control: from ‘moderate’ to high’. - Finding 12. Lack of follow-up from healthcare professionals: from ‘low’ to ‘moderate’ | We have   - elaborated on reasons for excluding potentially relevant studies in the manuscript text (line 220-224) and in a separate appendix (S3 Appendix Table of excluded studies). - revised the section “Criteria for considering studies for this review” to clarify that the **main topic of interest** in our qualitative evidence synthesis was to explore the views experiences of people with type 2 diabetes in maintaining lifestyle changes over time, defined as 12 months or more (line 97). - added six more studies to the review. The Results section is revised accordingly, including the PRISMA flowchart (fig 1). |
| **Reviewer#1:** There was no mention about the understanding of the languages of the study published other than English. | Reviewer #1 is correct in pointing out that our understanding of languages is not specified in the manuscript. When screening studies, we used Google Translate for abstracts written in other languages than English or the Scandinavian languages. We had a predefined plan to get studies translated into English where it was not possible to read the abstract or full-text in English. | We have specified the strategy for handling studies published in languages other than English or the Scandinavian languages during screening (line 135-38). The screening of records after updating the search was performed by CG and LVN, as specified in the manuscript (line 130). |
| **Reviewer#1:** There was also no mention about the data management during the process of evidence synthesis. | We agree with reviewer#1 that this is insufficiently described in the manuscript. We compiled relevant data from the studies, such as participant quotations or study authors’ summaries and descriptions of participants’ accounts, into an Excel spreadsheet. | We have specified how data from the individual studies were handled in the section “Data management, analysis and synthesis”, see line 154-56. We have also included the Excel file with the data supporting the review in a separate appendix (S2 Appendix: Supporting data from studies). |
| **Reviewer #2:** The area selected for review is good. The reviewers took effort to conduct the detailed review. It is mentioned articles of all languages selected. Not clear of the language expertise of reviewers. | Thank you for your feedback and question. Reviewer #1 is correct in pointing out that our understanding of languages is not specified in the manuscript. When screening studies, we used Google Translate for abstracts written in other languages than English or the Scandinavian languages. We had a predefined plan to get studies translated into English where it was not possible to read the abstract or full-text in English. | We have specified the strategy for handling studies published in languages other than English or the Scandinavian languages during screening (line XX). The screening of records after updating the search was performed by CG and LVN, as specified in the manuscript (line 130). |
| **Reviewer #2:** The six studies included in the final review is from 2003 to 2020, why did they take such a big gap? | The time span of the included studies from 2003 to 2020 reflects the available literature on the topic over the years. We have undertaken a comprehensive overview of the research and ensured inclusion of relevant literature across various time periods. In the revised manuscript, we have included a table of the reports that were assessed in full-text for eligibility and eventually excluded (n = 115). As shown in the table of excluded studies (S3 Appendix), numerous qualitative studies on patient experiences of living with T2D have been published over the years. The predominant reason for excluding studies was that they did not focus on maintaining lifestyle changes for 12 months or longer (i.e. the topic of interest in the studies did not comply with the topic of interest in our inclusion criteria).  When compiling the list of reasons for excluding studies from our review, we realized that one study (Wycherley et al., 2011, ref #44) excluded from the synthesis should have been included. The findings from this study are now integrated into the review.  Finally, we decided to update our literature search, as the previous search was conducted a year ago at the time of revision. This update led to the inclusion of five additional studies published between 2019 and 2024, bringing the total number of studies in the review to twelve. | We have   - elaborated on reasons for excluding potentially relevant studies in the manuscript text (line 220-24) and in a separate appendix (S3 Appendix Table of excluded studies). - added six more studies to the review, identified by revising the list of excluded studies and updating the search. The Results section, including the PRISMA flowchart (fig 2) is revised accordingly. |
| **Reviewer #2:** The review period is not clear from the article. | Thank you for pointing this out. As specified in the [review protocol](https://www.crd.york.ac.uk/prospero/display_record.php?ID=CRD42023395980) we started the review process in April 2022. We updated the literature search in November 2024, and extended the review with six additional studies before re-submitting the manuscript to PLOS ONE in March 2025. | We have revised the search date in the manuscript accordingly (line 118-19) |
| **Reviewer #2:** Different styles of articles compiled together to make the conclusion. | Unfortunately, we did not understand the second reviewer’s final comment. Can you please clarify? |  |
| **JOURNAL REQUIREMENTS** |  |  |
| We note that you have referenced (unpublished) on page 6, which has currently not yet been accepted for publication. Please remove this from your References and amend this to state in the body of your manuscript: (ie “Bewick et al. [Unpublished]”) as detailed online in our guide for authors  http://journals.plos.org/plosone/s/sub mission-guidelines#loc-reference-style | Thank you for notifying us about the format of this reference. The reference is a website, but we see that the format of the reference was incorrect. According to ICMJE sample references (39. Part of a homepage/Web site) it should be as follows:  Canadien Health Libraires Association [Internet]. Quebec: The Association; 2018 Jul 12 [cited 2022 Apr 26]. Recherche qualitative (Qualitative Research). Available from [https://extranet.santecom.qc.ca/wiki/!biblio3s/ doku.php?id=concepts:recherche-qualitative](https://extranet.santecom.qc.ca/wiki/!biblio3s/doku.php?id=concepts:recherche-qualitative) | The reference, a webpage, has been revised according the ICMJE standard (see line 775 / #24 in reference list). |
| As required by our policy on Data Availability, please ensure your manuscript or supplementary information includes the following:   - A numbered table of all studies identified in the literature search, including those that were excluded from the analyses. For every excluded study, the table should list the reason(s) for exclusion. | The PRISMA flow chart (Fig 1) illustrates the process from study identification to final inclusion. The literature search identified 4,101 unique records. Of these, 3,973 were excluded during the initial screening of titles and abstracts for not meeting our selection criteria. We assessed 128 full-text reports for eligibility, excluding 115. References and reasons for exclusion are provided in a separate appendix. | We provide a table of excluded studies with reasons for exclusion in S3 Appendix Table of excluded studies. |
| - A table of all data extracted from the primary research sources for the systematic review and/or meta-analysis. The table must include the following information for each study:  (a) Name of data extractors and date of data extraction (b) Confirmation that the study was eligible to be included in the review. (c) (All data extracted from each study for the reported systematic review and/or meta-analysis that would be needed to replicate your analyses. (d) (If data or supporting information were obtained from another source (e.g. correspondence with the author of the original research article), please provide the source of data and dates on which the data/information were obtained by your research group. | Thank you for bringing these issues to our attention. We have addressed them, except (d) as we did not obtain supporting information by contacting the authors of the original study reports. | We have included a table with extended information about the characteristics of each study (S2 Appendix: Characteristics and methodological limitations of included studies). The table includes information about (a), (b), and (c).  In addition, we have compiled all relevant data from the studies, such as participant quotations or study authors’ summaries and descriptions of participants’ accounts, into an Excel spreadsheet (see S2 Appendix: Supporting data from studies). |
| - If applicable for your analysis, a table showing the completed risk of bias and quality/certainty assessments for each study or outcome. Please ensure this is provided for each domain or parameter assessed. For example, if you used the Cochrane risk-of-bias tool for randomized trials, provide answers to each of the signalling questions for each study. If you used GRADE to assess certainty of evidence, provide judgements about each of the quality of evidence factor. This should be provided for each outcome. | Thank you for bringing these issues to our attention. We have addressed them. | We have included a table with extended information about the assessment of methodological limitations for each study (S2 Appendix: Characteristics and methodological limitations of included studies).  Our explanation of the GRADE-CERQual assessment for each review finding is found in the supplementary information:   - S5 Appendix S Summary of qualitative findings table - S6 Evidence Profiles |
| - An explanation of how missing data were handled. | Thank you for bringing this issue to our attention. We have addressed it. | We have added the following sentence to the manuscript: “We did not obtain supporting or missing information by contacting the authors of the original study reports.” (Data extraction, line 144-145). |
| - Changes to the reference list | We have included one reference to a systematic review in the background (ref 19) and an additional six studies in the results section (ref 42-47) | The reference list has been changed accordingly. |
